# Enzyme‐Like Synthetic Cleft for Light‐Driven Water‐Oxidation Catalysis Via an Oxide Relay Pathway

**DOI:** 10.1002/anie.3902517

**Published:** 2026-05-31

**Authors:** Daniel A. P. Friedewald, Gourab Das, Philipp H. Kirchner, Olga Anhalt, Deqi Tang, Sandra Luber, Florian Beuerle, Frank Würthner

**Affiliations:** ^1^ Institut für Organische Chemie Universität Würzburg Würzburg Germany; ^2^ Center for Nanosystems Chemistry (CNC) Universität Würzburg Würzburg Germany; ^3^ Department of Chemistry University of Zürich Zürich Switzerland; ^4^ Institut für Organische Chemie Universität Tübingen Tübingen Germany

**Keywords:** artificial photosynthesis, homogeneous catalysis, photocatalysis, ruthenium complexes, water oxidation

## Abstract

Harnessing functional groups in the outer coordination sphere to direct catalytic pathways is characteristic for enzymes, yet rather underexplored for man‐made catalysts. Here, following our earlier work on Ru(bda) water oxidation catalysts (bda = 2,2′‐bipyridine‐6,6′‐dicarboxylate), we demonstrate how a carboxyl‐functionalized Ru(bda)‐based macrocycle enables an oxide relay mechanism in light‐driven water oxidation via an O─O bond formation between carboxylate and Ru^V^ = O units positioned on opposing sides. Through a combination of photocatalytic water oxidation studies, NMR and single crystal analysis, and ^18^O‐labeling experiments, we provide evidence for the mechanistically distinct oxide relay pathway under photocatalytic conditions. Our findings underscore the role of second coordination sphere engineering in modulating reaction pathways and advancing molecular catalyst design.

## Graphical abstract

A carboxylic acid group introduced into the second coordination sphere of a Ru(bda) water oxidation catalyst forms a defined hydrogen bond within the catalytic cleft. This interaction locks the group in place and enables an intramolecular oxide relay pathway between Ru(V)═O and the carboxylate group, providing a distinct and efficient route for O─O bond formation in light‐driven water oxidation.

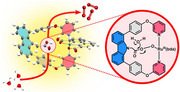

Man‐made transition metal catalysts are typically designed by tailored “first coordination sphere” ligands whose electronic and steric properties govern activity and (enantio‐) selectivity [1]. In contrast, natural metalloproteins also invoke the second coordination sphere provided by enzymatic clefts [2], thereby capitalizing on the entire space surrounding the active site. Inspired by water channels observed in x‐ray crystal structures of various metalloproteins [3, 4], including the oxygen‐evolving complex of photosystem II (OEC‐PSII) [5, 6, 7], chemists have, over the past decade, begun to take advantage of the second coordination sphere on catalytic activity. For the special case of the very demanding four‐electron oxidation of water into molecular oxygen, accomplished by OEC‐PSII, the enzymatic pocket obviously not only supports the diffusion of water molecules to the active site but in addition activates them for proton‐coupled electron transfer steps [8] taking place upon water oxidation. Based on this rationale Llobet and coworkers extended the equatorial 2,2’‐bipyridine‐6,6’‐dicarboxylate (bda) ligand utilized in Sun's highly successful Ru(bda) complexes [9, 10] to the respective terpyridine, thereby creating a dangling carboxylic acid group that supports the proton‐coupled electron transfer step significantly under electrocatalytic conditions (Figure 1a) [11]. At the same time our group introduced macrocyclic trinuclear architectures of Ru(bda) complexes and demonstrated the acceleration of the water nucleophilic attack (WNA) to the Ru^V^ = O species by water networks confined within the macrocycles under photocatalytic conditions [12, 13, 14]. More recently, we were even able to design an enzyme‐like pocket by incorporating a Ru(bda) subunit into a well‐defined macrocycle equipped with a bipyridine backbone [15]. Under acidic conditions, the protonation of the bipyridine unit established preorganized water substrates in front of the catalytic center, affording high TOF values upon oxidation with cerium ammonium nitrate (CAN) via the WNA mechanism. However, under neutral conditions in the photocatalytic mode with [Ru(bpy)_3_]^2+^ (bpy = 2,2′‐bipyridine) as photosensitizer, a switch to a slower bimolecular pathway involving the interaction of two metal‐oxo species (I2M mechanism) was observed, caused by deprotonation of the bipyridinium site and concomitant rotation of the open Ru site outside of the cavity. Accordingly, under neutral conditions, there was no advantage of the pocket design and the catalyst performed poorly (TOF values of 0.05–0.1 s^−1^), inferior to other available Ru(bda) complexes reported by our and other laboratories [10, 16, 17].

**FIGURE 1:**
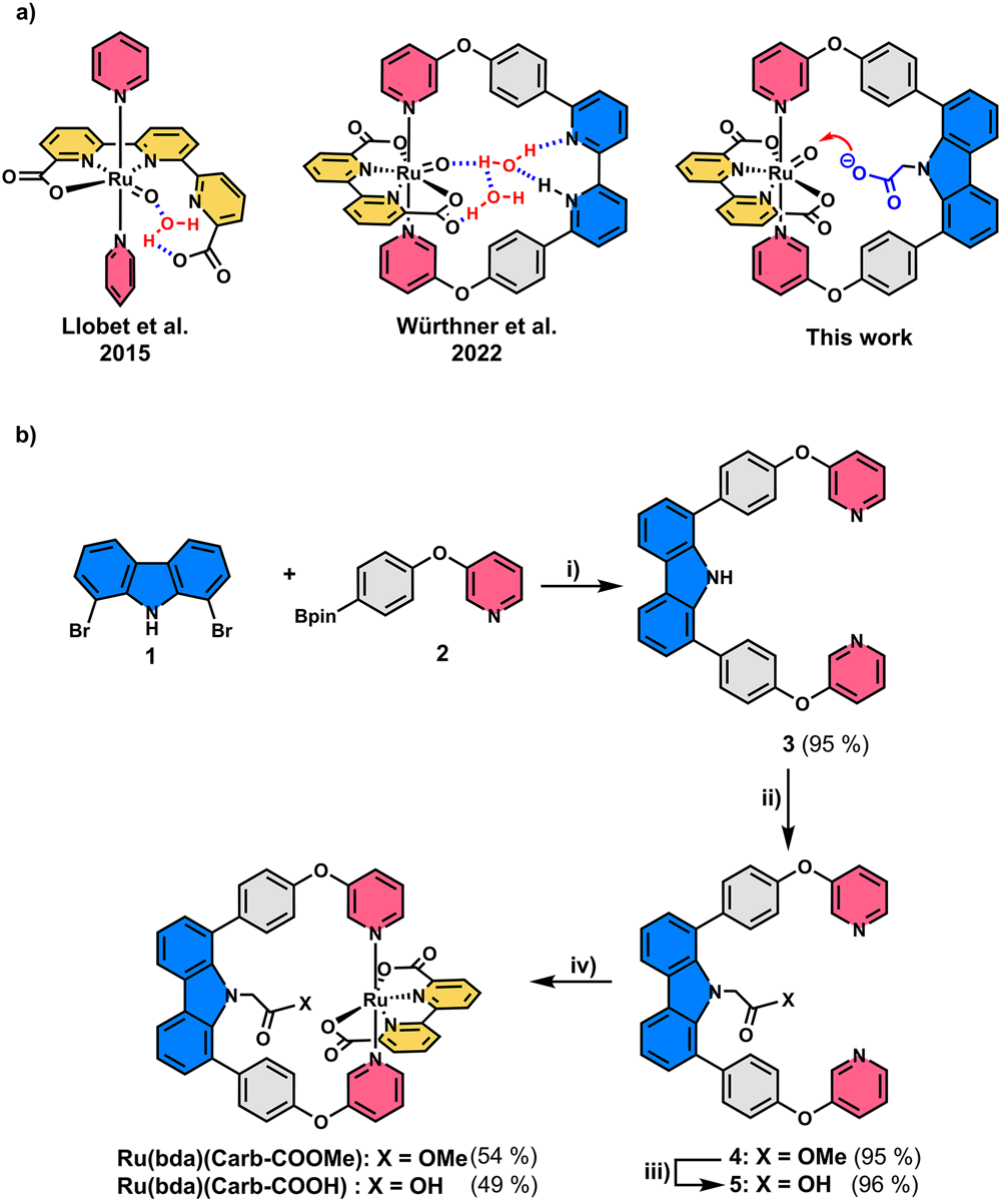
(a) Previous examples for Ru water oxidation catalysts with tailored nanoenvironments and concept of enzyme‐like pocket design for the oxide relay pathway. (b) Synthesis of molecular clefts **Ru(bda)(Carb‐COOMe)** and **Ru(bda)(Carb‐COOH)**. i) Na_2_CO_3_, Pd(PPh_3_)_4_, PhMe/H_2_O/EtOH, 16 h, 100°C; ii) Br‐CH_2_‐COOMe, NaH, DMF, 4 h, rt; iii) NaOH, EtOH/H_2_O, 16 h, 90°C; iv) [Ru(bda)(dmso)_2_], CHCl_3_/MeOH, 60°C, 16 h.

In general, water oxidation proceeds via two distinct mechanisms: the WNA and the I2M mechanism. The WNA mechanism involves the direct attack of water on a high‐valent Ru‐oxo species, leading to O─O bond formation. In contrast, the I2M mechanism entails the coupling of two Ru‐oxo species to form the O─O bond [18]. For the I2M mechanism, the self‐assembly of two subunits via π−π interactions or the incorporation of two catalytic units in close proximity within a single molecule have proven to be very effective for catalytic enhancement [19, 20, 21]. However, this bimolecular pathway is incompatible with low catalyst concentrations or the fixation of the catalyst to solid supports. As a possible resort to circumvent this problem, a mechanistic pathway analogous to the WNA mechanism, termed the “oxide relay pathway”, has been suggested through theoretical studies and subsequently confirmed experimentally under acidic conditions [11, 22, 23]. This pathway is characterized by a key intramolecular nucleophilic attack, wherein an oxygen atom from a pendant carboxylic acid group engages with the highly oxidized Ru^V^ ═ O species, replacing the conventional attack by an external water molecule. Hydrolytic cleavage of the percarboxylate species then leads to oxygen generation. In this communication, we adjust the design of our original molecular cleft [15] and recently reported structurally related ones based on dibenzo‐NSO‐heterocycles [24] by replacing the bipyridine backbone for a carbazole unit equipped with an additional carboxylic acid (**Ru(bda)(Carb‐COOH)**). NMR studies and single crystal x‐ray analysis reveal a well‐defined closed structure with the open Ru site facing into the cavity and the carbazole carboxylic acid forming a hydrogen bond to an oxygen of the opposing bda ligand. Photocatalytic measurements reveal a unique oxide relay pathway with significantly higher rate compared to the I2M mechanism observed for **Ru(bda)(Carb‐COOMe)**.

The novel molecular clefts **Ru(bda)(Carb‐COOH)** and **Ru(bda)(Carb‐COOMe)** were prepared according to the route depicted in Figure 1b starting from 1,8‐dibromocarbazole **1** and pinacol boronic ester **2** [24]. Coupling of these two compounds via a twofold Suzuki–Miyaura reaction gave ditopic ligand **3** in 95% yield. Alkylation of **3** afforded precursor **4** in 95% yield which could subsequently be hydrolyzed with excellent yields using sodium hydroxide in an ethanol/water mixture. Finally, by exchanging the weakly coordinated DMSO ligands of Ru precursor [Ru(bda)(dmso)_2_] by these bidentate ligands **Ru(bda)(Carb‐COOH)** and **Ru(bda)(Carb‐COOMe)** were obtained in 49% and 54% yield, respectively.

Both target molecules were fully characterized by NMR spectroscopy, mass spectrometry and single crystal x‐ray crystallography. Depending on the temperature, the ^1^H NMR spectra of the target compounds showed severe line broadening in the aromatic region (Figures S20 and S21), originating from dynamic rotation of the phenylene spacers within the macrocycle. At lower temperatures, these rotations are restricted leading to distinct signals attributable to a specific predominant conformation (Figures 2,  S12 and S13). Thus, the typical AA'BB' pattern of the phenylene units (grey color) can no longer be seen in the ^1^H NMR spectrum of **Ru(bda)(Carb‐COOH)** as the two signals split up into four individual signals indicating the breaking of symmetry. Two of the three proton signals of the Ru(bda) backbone (yellow color) also split up into individual signals (Figure 2). When comparing the proton signals of the pyridine unit in the final compound and in the precursor molecule, a significant difference in chemical shift can be made out for the green‐labelled *ortho* proton of the pyridine unit. Although this proton resonates at 8.25 ppm in precursor **5**, it is significantly upfield shifted to 6.15 ppm upon coordination of the Ru(bda) moiety in compound **Ru(bda)(Carb‐COOH)**. This is in stark contrast to the other *ortho* proton labelled in purple, whose signal shift is less pronounced with a minor downfield shift (8.36 to 8.59 ppm). The significant upfield shift observed for the green‐labelled proton signal can be attributed to the orientation of the Ru(bda) unit, which is rotated outside of the cavity. This favored orientation can be explained by the steric demand of the alkyl substituent at the carbazole nitrogen atom that prohibits the rotation of the bda ligand into the pocket as observed in our earlier cleft design [14] with 2,2’‐bypyridine instead of alkylated carbazole. Based on very similar ^1^H NMR results for **Ru(bda)(Carb‐COOMe)** (Figure S22) we conclude that the structures of both target molecules are similar affording the desired enzyme‐like catalytic pocket.

**FIGURE 2:**
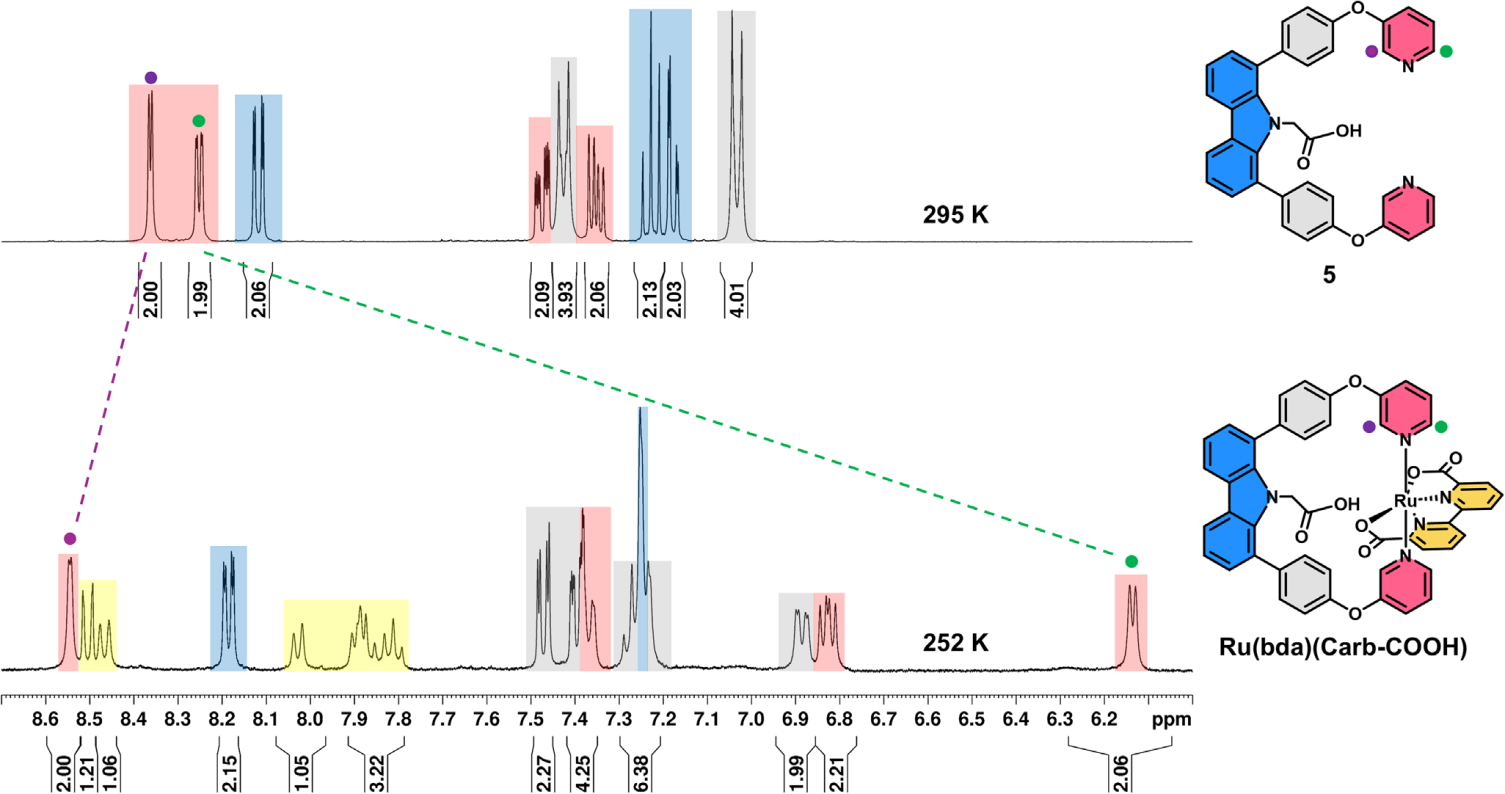
^1^H NMR spectra (4:1 CD_2_Cl_2_/MeOD, 400 MHz, variable temperatures) of **5** (top) and **Ru(bda)(Carb‐COOH)** (bottom) with color coded signals according to the molecular structures on the right. The colours correspond to bda (yellow), axial pyridine units (red), phenylene group (grey) and carbazole moiety (blue).

For further structural insights, single crystals of **Ru(bda)(Carb‐COOH)** and **Ru(bda)(Carb‐COOMe)** were grown by vapor diffusion of diethyl ether into a concentrated solution of the respective compound in a 4:1 DCM/MeOH mixture. The single‐crystal X‐ray structures of both target molecules are depicted in Figure 3. Remarkably, both structures found in the solid state perfectly match up with the proposed conformations deduced from ^1^H NMR measurements in solution. Both macrocycles exhibit a 6‐coordinated Ru complex with a highly distorted octahedral configuration, characterized by obtuse O─Ru─O angles of 121.8° and 122.0°. This distortion is further enhanced by the rigid structure of the macrocycles, which induces a deviation of the usually linear N_ax_─Ru─N_ax_ bond angle to a reduced value of 171.9° for both molecules. However, comparison of the orientation of the various carbonyl moieties reveals a notable difference. Whilst the carboxylic ester unit of **Ru(bda)(Carb‐COOMe)** is pointing outside of the cavity (with only minor interaction between the ester CH_3_ group and the equatorial bda ligand), the acid group of **Ru(bda)(Carb‐COOH)** is fixed inside by means of a hydrogen bond to one of the carboxylic acid groups of the equatorial bda ligand. A bond length of 1.79 Å and an almost ideal linear configuration with an O─H─O angle of 177.5° indeed indicates a rather strong H─bond.

**FIGURE 3:**
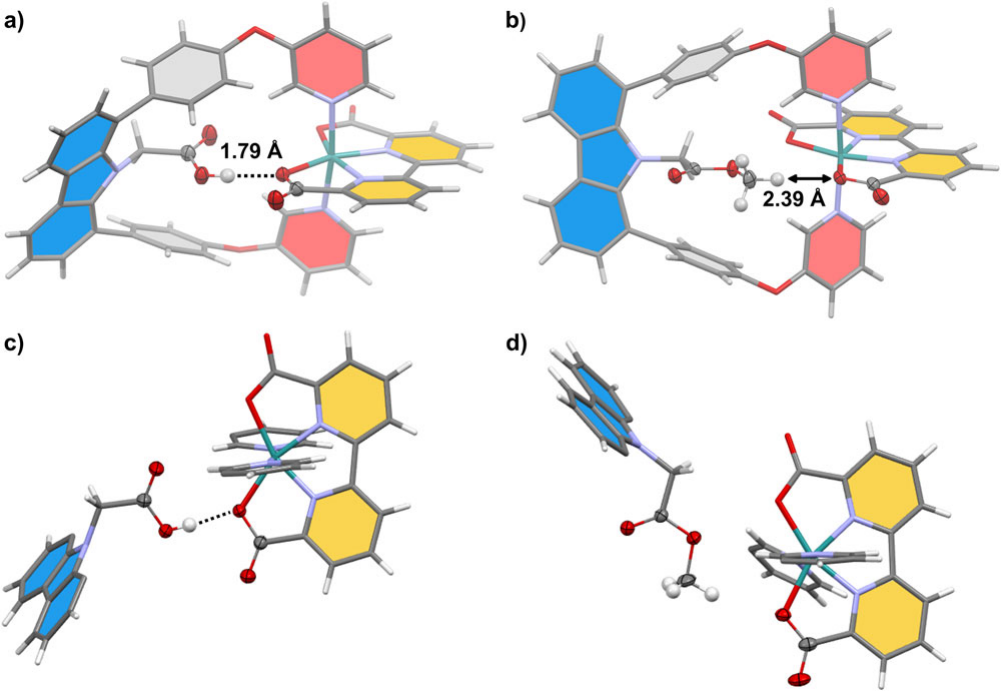
Top: Side view of the molecular structures of (a) **Ru(bda)(Carb‐COOH)** and (b) **Ru(bda)(Carb‐COOMe)** according to single‐crystal x‐ray analyses with indicated intramolecular hydrogen bond for **Ru(bda)(Carb‐COOH)**. Solvent molecules omitted for clarity. Ellipsoids set at 50% probability. Bottom: Top view of the molecular structures of (c) **Ru(bda)(Carb‐COOH)** and (d) **Ru(bda)(Carb‐COOMe)** according to single‐crystal x‐ray analyses. Solvent molecules and inner phenyl ring atoms omitted for clarity. Ellipsoids set at 50% probability.

The performance of both macrocycles as catalysts for light‐driven water oxidation were investigated using a three‐component system in 50 mM phosphate buffered CH_3_CN/H_2_O 4:6 mixtures at pH 7 with [Ru(bpy)_3_Cl_2_] (*c* = 1.5 mM) as photosensitizer and Na_2_S_2_O_8_ (*c* = 37 mM) as sacrificial electron acceptor (Figure S1). Irradiation was achieved using a xenon arc lamp equipped with a solar filter operating at a power of 100 mW cm^−2^. Interestingly, whilst **Ru(bda)(Carb‐COOMe)** showed a second‐order dependency of O_2_ evolution on the WOC concentration with a TOF of 0.04–0.16 s^−1^, **Ru(bda)(Carb‐COOH)** exhibited a linear dependency with a TOF value of 0.23 s^−1^ (Figure 4a) [25].

**FIGURE 4:**
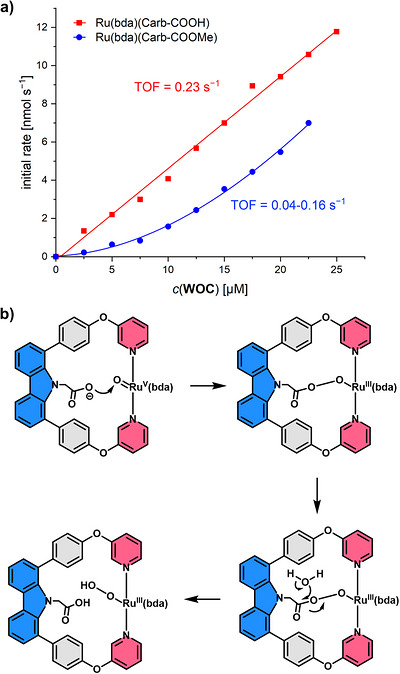
(a) Photochemical water oxidation catalysis with **Ru(bda)(Carb‐COOMe)** and **Ru(bda)(Carb‐COOH)** as WOCs and [Ru(bpy)_3_Cl_2_] (*c* = 1.5 mM) as photosensitizer in CH_3_CN/H_2_O 4:6 (pH 7, 50 mM phosphate buffer), *c*(PS) = 1.5 mM, *c*(Na_2_S_2_O_8_) = 37 mM. Plot of initial oxygen evolution against WOC concentration. (b) Proposed oxide relay pathway mechanism for **Ru(bda)(Carb‐COOH)**.

These disparate dependencies of the initial rate on Ru catalyst concentration have to be attributed to different mechanisms by which the macrocycles operate. With the second‐order kinetics **Ru(bda)(Carb‐COOMe)** operates via the bimolecular I2M pathway (Figure S2), i.e. two Ru^V^ = O species forming bimolecular oxygen via a [Ru^V^ = O⋯O = Ru^V^] intermediate [26], while **Ru(bda)(Carb‐COOH)** follows a unimolecular mechanism. To gain further insights into the mechanistic pathway, photocatalytic measurements with D_2_O instead of H_2_O were performed for both catalysts (Figures S7 and S8). Remarkably, despite of the linear kinetics, no kinetic isotope effect was observed for **Ru(bda)(Carb‐COOH)** as expected for the commonly with linear kinetics associated WNA mechanism that is characterized by a rate‐determining proton‐coupled electron transfer step (Figure S2). This observation leads to the conclusion that a proton transfer is not involved in the rate‐determining step, thereby ruling out the conventional WNA mechanism. As mentioned above, the crystal structure of **Ru(bda)(Carb‐COOH)** revealed the close proximity of the pendant carboxylic acid group to the ruthenium center, placing it well within reach for intramolecular interaction with the high‐valent Ru‐oxo species. Such a spatial arrangement closely resembles systems reported in the literature in which dangling carboxylic acid groups near the active site promote an alternative oxide‐relay pathway [22, 23, 27]. In this mechanistic pathway, the metal‐oxo species is not attacked by a water molecule, but instead the oxygen atom of the adjacent carboxylate group attacks the high valent metal‐oxo species in an intramolecular fashion (Figure 4b). The resulting percarboxylate species is then cleaved by WNA at the carbonyl moiety and subsequently O_2_ is generated. The observed TOF values remained unchanged at pH 9 and 10 (Figures S5 and S6), corroborating that the rate‐determining step is the nucleophilic attack of the carboxylate species.

To provide further evidence for the proposed mechanism, the standard photocatalytic experiments were performed in ^18^O‐labeled water in place of regular water. If the molecule follows the oxide‐relay mechanism proposed in Figure 4b, postcatalytic mass spectrometry should show the incorporation of the heavier oxygen isotope into the catalyst, evidenced by a mass increase of two units [23]. This is exactly what we observed in the MALDI‐TOF spectrum of the post‐catalytic sample that exhibits a molecular ion peak at *m*/*z* of 909.133, exactly two‐mass‐units upshifted to the mass to *m*/*z* 907.104 measured after catalysis using regular water (Figure S28). The isotope pattern obtained from the experiment consists of labeled ^18^O and earth abundant ^16^O complexes, with a simulated percentage ratio of [**Ru(bda)(Carb‐CO^16^OH)**]^+^: [**Ru(bda)(Carb‐CO^18^OH)**]^+^ = 1:1. A control experiment using ^18^O‐labeled water without photosensitizer or sacrificial electron acceptor did not afford the ^18^O‐labeled catalyst which ensures that the incorporation of ^18^O atoms does not occur due to exchange of hydroxy groups of the carboxylate with the solvent. A stirred catalyst solution overnight likewise did not show any exchange. Thus, mass spectrometric analysis clearly indicates that oxygen atoms of water are incorporated into the catalyst during the catalytic cycle, fully consistent with the proposed oxide‐relay pathway. Additional support for the proposed oxide‐relay mechanism comes from density functional theory (DFT) calculations. The calculated electronic barriers for the intramolecular O─O bond formation between the pendant carboxylate and the Ru^V^ = O unit, and for the subsequent nucleophilic attack of water on the percarboxylate intermediate, are 76 and 84 kJ mol^−1^ respectively (Figure S29). These values are consistent with a rapid, unimolecular process occurring at room temperature.

In this work, a Ru(bda) water oxidation catalyst was engineered for an unprecedented oxide relay pathway via a percarboxylate species by positioning a carboxylic acid unit straight in front of the Ru^V^ = O species. Related second coordination sphere effects are commonly observed in enzymatic clefts but still rather uncommon in the design of man‐made transition metal catalysts. By means of our enzyme‐like molecular cleft design, a mechanistic switch from a bimolecular to the more desired unimolecular pathway has been achieved under photocatalytic conditions for **Ru(bda)(Carb‐COOH)** as confirmed by kinetic studies, kinetic isotope effects as well as isotope labelling. In contrast, the **Ru(bda)(Carb‐COOMe)** derivative, which lacks a pendant carboxylic acid group, shows none of these features, underscoring the essential role of the carboxylate functionality in enabling the oxide‐relay mechanism. NMR spectroscopy and X‐ray analysis provided unequivocal evidence for the perfect positioning of the carboxylic acid functional group in front of the ruthenium center. Such advanced supramolecular catalyst design principles should be likewise considered for other catalysts, taking advantage of the whole space around the transition metal [28], as they might not only be useful for the acceleration of rates as in the given example but also enable improved substrate selectivity and even control of chirality.

## Author Contributions

**Daniel A. P. Friedewald**: investigation, writing – original draft, methodology, validation, visualization, formal analysis, data curation. **Gourab Das**: investigation, writing – original draft, methodology, formal analysis. **Philipp H. Kirchner**: investigation, methodology, formal analysis. **Olga Anhalt**: Investigation, methodology, validation, formal analysis. **Deqi Tang**: investigation, writing – original draft, software, data curation, validation. **Sandra Luber**: writing – review and editing, validation, data curation, supervision. **Florian Beuerle**: writing – review and editing, supervision. **Frank Würthner**: conceptualization, funding acquisition, writing – review and editing, project administration, resources, supervision.

## Conflicts of Interest

The authors declare no conflicts of interest.

## Supporting information




**Supporting File**: The authors have cited additional references within the Supporting Information [29, 30, 31, 32, 33, 34, 35, 36, 37, 38, 39, 40, 41, 42, 43, 44, 45, 46, 47, 48, 49, 50, 51, 52, 53, 54, 55, 56, 57, 58, 59, 60, 61, 62, 63, 64].

## Data Availability

The data that support the findings of this study are openly available in Zenodo at https://doi.org/10.5281/zenodo.16902537. Accession Codes CCDC2481884 and CCDC2481885 contain the supplementary crystallographic data for this paper. These data can be obtained free of charge via www.ccdc.cam.ac.uk/data_request/cif, or by emailing data_request@ccdc.cam.ac.uk, or by contacting The Cambridge Crystallographic Data Centre, 12 Union Road, Cambridge CB21EZ, UK; fax: +441223 336033.
