# Supplementary material for: Enzyme‐Like Synthetic Cleft for Light‐Driven Water‐Oxidation Catalysis Via an Oxide Relay Pathway
Source: Angew Chem Int Ed Engl. 2026 May 31;65(29):e3902517. doi: 10.1002/anie.3902517 (PMC13360865; doi:10.1002/anie.3902517)
Supplement: Supplementary file 1 — Supporting File: The authors have cited additional references within the Supporting Information [29, 30, 31, 32, 33, 34, 35, 36, 37, 38, 39, 40, 41, 42, 43, 44, 45, 46, 47, 48, 49, 50, 51, 52, 53, 54, 55, 56, 57, 58, 59, 60, 61, 62, 63, 64]. [file ANIE-65-e3902517-s001.pdf]

Supporting Information

Enzyme-like Synthetic Cleft for Light-driven Water-Oxidation Catalysis via an Oxide Relay Pathway

Daniel A. P. Friedewald,[a] Gourab Das,[a] Philipp H. Kirchner,[a] Olga Anhalt,[b] Deqi Tang,[c] Sandra Luber,[c] Florian Beuerle,[d] Frank Würthner[a,b]*

[a] Institut für Organische Chemie, Universität Würzburg, Am Hubland, 97074 Würzburg (Germany)

[b] Center for Nanosystems Chemistry (CNC), Universität Würzburg, Theodor-Boveri-Weg, 97074 Würzburg (Germany)

[c] Department of Chemistry, University of Zürich, 8057 Zürich (Switzerland)

[d] Institut für Organische Chemie, Universität Tübingen, Auf der Morgenstelle 18, 72076 Tübingen (Germany)

* Email: frank.wuerthner@uni-wuerzburg.de

Table of Contents

Materials and Methods 1
Experimental Procedures 4
Single Crystal X-Ray analysis 7
Photocatalytic Water Oxidation 8
Kinetic Isotope Effect 11
Electrochemistry 12
VT-NMR spectra 14
NMR spectra 15
HR mass spectra 20
Theoretical Calculations 23
References 24

Materials and Methods

General

All chemicals and solvents were purchased from commercial sources and used without further purification, unless stated otherwise. 2,2'-Biypyridine-6,6'-dicarboxylic acid (bda)[1], $[RuCl_2(dmso)_4]$[2,3], $[Ru(bda)(dmso)_2]$[4], 1,8-Dibromocarbazole[5] and 3-(4-(4,4,5,5-tetramethyl-1,3,2-dioxaborolan-2-yl)phenoxy)pyridine[6] were synthesized according to literature procedures. Reactions with air or moisture sensitive reagents were performed under a nitrogen atmosphere using standard Schlenk techniques. Crude isolated compounds were further purified by column chromatography on silica gel (60M, 0.04-0.063 mm, Macherey-Nagel) with freshly distilled solvents. Flash column chromatography was performed using an automated flash purification system PuriFlash 420 (Interchim) using pre-packed silica gel columns (Interchim) with a grain size of 30 µm and distilled solvents. All experiments in aqueous solutions were performed either in phosphate buffer pH 7 (Honeywell) or deionized water obtained from a Purelab Classic water purification system (ELGA). The ^{18}O-labelled water (97%) was purchased from Deutero GmbH, Germany.

NMR spectroscopy

^{1}H NMR and proton decoupled ^{13}C NMR spectra were recorded on a Bruker Avance III HD 400 spectrometer at 298 K as well as 252 K. Chemical shifts (δ) are reported in parts per million (ppm) relative to tetramethylsilane and referenced internally to the residual solvent signal.[7] The respective coupling constants (J) are given in Hertz (Hz). To describe signal multiplicities, the following abbreviations were applied: s = singlet, d = doublet, t = triplet and m = multiplet. In addition, 2D NMR spectra (COSY, NOESY, HSQC and HMBC) were recorded to allow a correct assignment of 1D NMR spectra of novel compounds. For the final compounds **Ru(bda)(Carb-COOMe)** and **Ru(bda)(Carb-COOH)** no ^{13}C NMR spectra could be recorded due to solubility issues at the required lower temperatures.

Mass spectrometry

High-resolution electrospray ionization (HR-ESI) measurements were obtained on an ESI MicrOTOF focus mass spectrometer (Bruker Daltonics GmbH). MALDI TOF (Matrix-assisted laser desorption/ionisation time-of-flight) mass spectrometry measurements were acquired on a Bruker Daltonics UltrafleXtreme mass spectrometer using DCTB (*trans*-2-[3-(4-*tert*-butylphenyl)-2-methyl-2-propenylidene] malononitrile) as matrix.

Electrochemistry

Cyclic and differential pulse voltammetry (CV and DPV) experiments were performed on a BAS Cell Stand C3 (BAS Epsilon) using a glassy carbon disc as working electrode, a Pt wire as counter electrode and Ag/AgCl (3 M KCl) as reference electrode. The measurements were conducted at a scan rate of 100 mV s^{-1} at 298 K. All experiments were measured in homogenous phase in a 1:1 mixture of 2,2,2-trifluorethanol/H_2O (pH 7, 50 mM phosphate buffer). All measured potentials are reported vs. normal hydrogen electrode (NHE) by addition of +0.21 V.[8] The respective Pourbaix diagrams were created based on multiple DPV measurements in phosphate buffered aqueous solution at different pH values (I = 0.1 M) in presence of 50% TFE.

Single crystal X-ray analysis

Single crystals of **Ru(bda)(Carb-COOMe)** and **Ru(bda)(Carb-COOH)** were grown by slow diffusion of diethyl ether in a DCM/MeOH (1:1) solution of the respective complex stored in the fridge. Measurements were performed at 100 K on Bruker's D8 Quest Kappa diffractometer with a Photon II CPAD as detector. The diffraction data were processed with the help of the APEX3 and APEX4 program packages. The structures were solved with the help of the SHELXT[9] software and subsequently further processed using Fourier techniques, where SHELX[10] software was used. The PLATON SQUEEZE program was used to remove disordered solvent molecules.[11,12]

Photocatalytic water oxidation

Photocatalytic water oxidation experiments were carried out using an Oxygraph Plus Clark-electrode (Hansatech Instruments Ltd.) for oxygen detection. Irradiation of the samples was performed with a Xenon lamp (Newport, 150 W, calibrated to 100 mW cm^{-1}) equipped with a UV-cutoff filter (400 nm). Irradiation was calibrated to 100 mW cm^{-2} using a PM200 optical power meter equipped with an S121C sensor (Thorlabs) and a CCS200/M wide-range spectrometer (Thorlabs). For each measurement, a stock solution containing $[Ru(bpy)_3]Cl_2$ as the photosensitizer (PS) and $Na_2S_2O_8$ as the sacrificial electron acceptor was prepared in the dark using an aqueous mixture (pH 7, phosphate buffer) with acetonitrile as the organic co-solvent (c($Na_2S_2O_8$= 37 mM, c($[Ru(bpy)_3]Cl_2$) = 1.5 mM). A portion of this stock solution was combined with the catalyst solution in a transparent, water-cooled reaction chamber maintained at 20 °C, while kept in the dark. Irradiation commenced 45 seconds after mixing to ensure adequate equilibration. The turnover number (TON) was calculated by dividing the maximum amount of evolved oxygen during the experiment by the amount of catalyst used. The turnover frequency (TOF) was determined by dividing the initial rate of oxygen evolution

by the catalyst concentration. Initial rates were obtained via linear regression of the first 10 seconds of oxygen evolution following light irradiation.

The ^{18}O isotope-labeling studies were carried out by suspending small amounts of Ru(bda)(Carb-COOH) (500 µg, 551 nmol) in pure $H_2{}^{18}O$ (500 µL), together with the photosensitizer $[Ru(bpy)_3]Cl_2$ (10 eq.) and the sacrificial electron acceptor $Na_2S_2O_8$ (50 eq.). The suspension was irradiated for 60 seconds using the Xenon lamp employed in the photocatalytic water oxidation experiments. The incomplete incorporation of ^{18}O into the catalyst can be explained by its low solubility in pure water, which prevented complete participation of all catalyst molecules in the reaction. The use of MeCN as co-solvent was avoided, as it led to fragmentation of the complex during the subsequent mass spectrometry analysis; therefore, the aqueous method described above was chosen.

Computational Details

Nudged elastic band (NEB) calculations using Kohn-Sham density functional theory were performed to predict the electronic energies related to the energetics of the oxide relay mechanism for **Ru(bda)(Carb-COOH)**. The attacking H_2O molecule was initially positioned in proximity to the O-O bond. The product state was constructed by coordinating one hydrogen atom from H_2O to the carboxylate oxygen (COO^-) and the remaining hydroxyl group to the oxo center. A free-end NEB calculation was subsequently employed to concurrently optimize the reaction pathway and the terminal states. Following several iterations, local minima were identified along the energy profile; these structures were then extracted as refined reactant and product states for a final, converged NEB production run. The climbing image nudged elastic band (CI-NEB) method was employed as implemented in the ORCA 6.1.0 software.[13-15] The B3LYP functional together with Triple-Zeta Valence Polarization (def2-TZVP) basis set were used as in our earlier works.[16-22] The Grimme D3 dispersion corrections were used.[23,24] The Conductor-like Polarizable Continuum Model (CPCM) implicit solvent for water was used to approximate the solvent effect.[25] Our calculations comparing the electronic energies of **Ru(bda)(Carb-COOH)** with/without oxo show that the low spin state (spin multiplicity 2) has a lower electronic energy than the high spin state (spin multiplicity 4). Therefore, the low spin state was considered in the subsequent calculations, which is also generally recognized as the stable spin state for Ru(bda) catalysts.[26-28] In the NEB calculations, energy-weighted spring constants scaling from lower-bound of 0.01 a.u. to an upper-bound of 0.2 a.u. were used.[29] The convergence thresholds of 0.002 and 0.001 a.u. for the maximum component of the atomic force acting on the climbing image (max(|FCI|)) and the root mean squared atomic force acting on the climbing image (RMS(FCI)) were set, respectively.

Experimental Procedures

1,8-bis(4-(pyridin-3-yloxy)phenyl)-9H-carbazole (3)

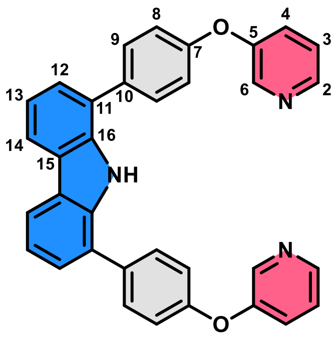

3

1,8-Dibromocarbazole **1** (1.21 g, 3.72 mmol, 1.0 eq.), 3-(4-(4,4,5,5-tetramethyl-1,3,2-dioxaborolan-2-yl)phenoxy)pyridine **2** (2.43 g, 8.19 mmol, 2.2 eq.), Na_2CO_3 (3.16 g, 29.8 mmol, 8.0 eq.) and tetrakis(triphenylphosphine)palladium (430 mg, 372 µmol, 0.1 eq.) were dissolved in a 4:2:1 mixture of toluene/water/ethanol (53 mL) and stirred at 100 °C for 16 h. Afterwards, the solvent was removed under reduced pressure. The residue was taken up in water and extracted with dichloromethane. The combined organic phases were dried over $MgSO_4$, and the solvent removed under reduced pressure. The crude product was purified by flash chromatography on silica gel using mixtures of dichloromethane and ethyl acetate (9:1 to 7:3) as eluent.

Yield: 1.80 g (3.55 mmol, 95 %) of an off-white solid.

^{1}H NMR (400 MHz, CD_2Cl_2/MeOD 4:1, 295 K): δ = 8.37 (dd, ^4J = 2.8 Hz, ^5J = 0.6 Hz, 2H, H-6), 8.30 (dd, ^3J = 4.7 Hz, ^4J = 1.4 Hz, 2H, H-2), 8.09 (dd, ^3J = 7.8 Hz, ^4J = 1.2 Hz, 2H, H-14), 7.71–7.66 (AA', 4H, H-9), 7.46–7.41 (m, 4H, H-4, H-12), 7.38–7.30 (m, 4H, H-3, H-13), 7.20–7.15 (BB', 4H, H-8) ppm.

^{13}C NMR (101 MHz, CD_2Cl_2/MeOD 4:1, 295 K): δ = 156.2, 154.6, 144.4, 141.2, 137.7, 135.5, 130.3, 126.8, 126.5, 125.2, 124.8, 124.5, 120.7, 120.2, 120.0 ppm.

HRMS (ESI-TOF, positive mode, $CHCl_3$/MeCN 1:1): *m/z* calculated for $C_{34}H_{24}N_3O_2$ $[M+H]^+$: 506.1863; found 506.1858.

1-(1,8-bis(4-(pyridin-3-yloxy)phenyl)-carbazol-9-yl)-2-methoxyethan-1-one (4)

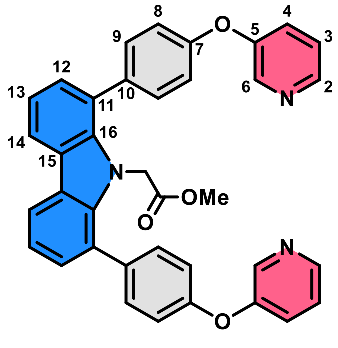

4

3 (200 mg, 396 µmol, 1.0 eq.) was dissolved in dry DMF (6 mL) and NaH (60 % wt in mineral oil, 31.1 mg, 779 µmol, 2.0 eq.) was added. Methyl bromo acetate (45.7 µL, 483 µmol, 1.22 eq.) was added and the yellow solution stirred at room temperature for 4 h and quenched by addition of H_2O. The crude mixture was extracted with dichloromethane, the combined organic phases were dried over $MgSO_4$, and the solvent removed under reduced pressure. The crude product was purified by flash chromatography on silica gel using a mixture of dichloromethane and ethyl acetate (4:6) as eluent.

Yield: 216 mg (374 µmol, 95 %) of an off-white solid.

^{1}H NMR (400 MHz, CD_2Cl_2/MeOD 4:1, 295 K): δ = 8.35 (dd, ^4J = 2.7 Hz, ^5J = 0.7 Hz, 2H, H-6), 8.30 (dd, ^3J = 4.5 Hz, ^4J = 1.6 Hz, 2H, H-2), 8.15 (dd, ^3J = 7.6 Hz, ^4J = 1.4 Hz, 2H, H-14), 7.43–7.34 (m, 8H, H-3, H-4, H-9), 7.30 (t, ^3J = 7.5 Hz, 2H, H-13), 7.23 (dd, ^3J = 7.3 Hz, ^4J = 1.4 Hz, 2H, H-12), 7.09–7.04 (m, 4H, H-8), 4.32 (s, 2H, CH_2), 3.32 (s, 3H, CH_3) ppm.

^{13}C NMR (101 MHz, CD_2Cl_2/MeOD 4:1, 295 K): δ = 170.0, 156.1, 154.8, 144.3, 141.0, 139.5, 136.4, 131.8, 129.7, 126.5, 125.8, 125.2, 120.3, 119.9, 119.3, 48.9 ppm.

HRMS (ESI-TOF, positive mode, $CHCl_3$/MeCN 1:1): *m/z* calculated for $C_{37}H_{28}N_3O_4$ $[M+H]^+$: 578.2074; found 578.2090.

2-(1,8-bis(4-(pyridin-3-yloxy)phenyl)-carbazol-9-yl)acetic acid (5)

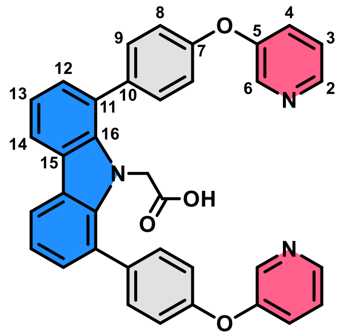

5

4 (90.0 mg, 155 µmol, 1.0 eq.) and NaOH (31.2 mg, 779 µmol, 5.0 eq.) were dissolved in a 1:1 H_2O/EtOH mixture (5 mL) and stirred at 90 °C for 16 h. EtOH was removed under reduced pressure and the mixture neutralized (pH 5) with 1 M HCl. The precipitate was collected, washed with H_2O and dried under vacuum.

Yield: 87.8 mg (149 µmol, 96 %) of a white solid.

^{1}H NMR (400 MHz, CD_2Cl_2/MeOD 4:1, 295 K): δ = 8.36 (d, ^4J = 2.8 Hz, 2H, H-6), 8.25 (dd, ^3J = 4.7 Hz, ^4J = 1.3 Hz, 2H, H-2), 8.12 (dd, ^3J = 7.5 Hz, ^4J = 1.4 Hz, 2H, H-14), 7.47 (ddd, ^3J = 8.5 Hz, ^4J = 2.8 Hz, ^4J = 1.3 Hz, 2H, H-4), 7.45–7.40 (AA', 4H, H-9), 7.35 (dd, ^3J = 8.5 Hz, ^4J = 4.7 Hz, 2H, H-3), 7.23 (t, ^3J = 7.4 Hz, 2H, H-13), 7.17 (dd, ^3J = 7.4 Hz, ^4J = 1.4 Hz, 2H, H-12), 7.06–7.00 (BB', 4H, H-8), 4.16 (s, 2H, CH_2) ppm.

^{13}C NMR (101 MHz, CD_2Cl_2/MeOD 4:1, 295 K): δ = 175.3, 155.7, 155.0, 144.0, 141.0, 140.3, 137.3, 131.7, 129.5, 126.6, 126.3, 125.2, 125.1, 119.5, 119.4, 119.2, 51.3 ppm.

HRMS (ESI-TOF, positive mode, $CHCl_3$/MeCN 1:1): *m/z* calculated for $C_{36}H_{26}N_3O_4$ $[M+H]^+$: 564.1918; found 564.1921.

Ru(bda)(Carb-COOMe)

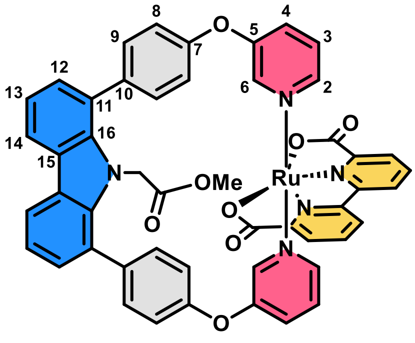

Ru(bda)(Carb-COOMe)

4 (104 mg, 180 µmol, 1.0 eq.) and $[Ru(bda)(DMSO)_2]$ (99.0 mg, 198 µmol, 1.1 eq.) were dissolved in a mixture of degassed methanol (35 mL) and chloroform (35 mL) and heated at 65 °C for 16 h. After cooling to room-temperature, the solvent was removed under reduced pressure and the crude product was purified by column chromatography on silica gel using a mixture of dichloromethane and methanol (96:4) as eluent.

Yield: 90.0 mg (97.8 µmol, 54 %) of a red solid.

^{1}H NMR (400 MHz, CD_2Cl_2/MeOD 4:1, 252 K): δ = 8.48 (d, ^4J = 2.6 Hz, 2H, H-6), 8.44–8.34 (m, 2H, H_{bda}), 8.19 (dd, ^3J = 7.6 Hz, ^4J = 1.5 Hz, 2H, H-14), 8.03–7.70 (m, 4H, H_{bda}), 7.54–7.49 (m, 2H, H-9), 7.38 (ddd, ^3J = 8.4 Hz, ^4J = 2.6 Hz, ^4J = 1.1 Hz, 2H, H-4), 7.34–7.29 (m, 2H, H-9) 7.28 (t, ^3J = 7.5 Hz, 2H, H-13), 7.23 (dd, ^3J = 7.2 Hz, ^4J = 1.5 Hz, 2H, H-12), 7.20–7.15 (m, 2H, H-8), 7.00–6.94 (m, 2H, H-8), 6.82 (dd, ^3J = 8.4 Hz, ^4J = 5.5 Hz, 2H, H-3), 6.12 (d, ^3J = 5.5 Hz, 2H, H-2), 4.55 (s, 2H, CH_2), 3.78 (s, 3H, CH_3) ppm.

HRMS (ESI-TOF, positive mode, $CHCl_3$/MeCN 1:1): *m/z* calculated for $C_{49}H_{33}N_5O_8Ru$ $[M]^+$: 921.1373; found 921.1384.

Ru(bda)(Carb-COOH)

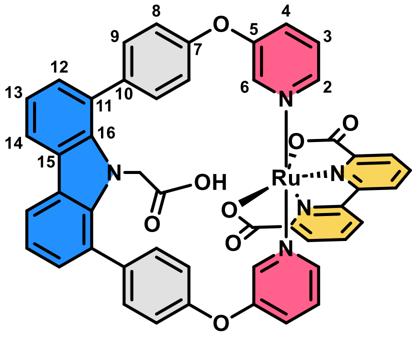

Ru(bda)(Carb-COOH)

5 (145 mg, 257 µmol, 1.0 eq.) and $[Ru(bda)(DMSO)_2]$ (142 mg, 283 µmol, 1.1 eq.) were dissolved in a mixture of degassed methanol (40 mL) and chloroform (40 mL) and heated at 65 °C for 16 h. After cooling to room-temperature, the solvent was removed under reduced pressure and the crude product was purified by column chromatography on silica gel using a mixture of dichloromethane and methanol (96:4) as eluent.

Yield: 114 mg (126 µmol, 49 %) of a red solid.

^{1}H NMR (400 MHz, CD_2Cl_2/MeOD 4:1, 252 K): δ = 8.59 (d, ^4J = 2.6 Hz, 2H, H-6), 8.53–8.44 (m, 2H, H_{bda}), 8.18 (dd, ^3J = 7.0 Hz, ^4J = 2.1 Hz, 2H, H-14), 8.05–7.77 (m, 4H, H_{bda}), 7.50–7.45 (m, 2H, H-9), 7.42–7.36 (m, 4H, H-4, H-9), 7.33–7.26 (m, 6H, H-8, H-12, H-13), 6.91–6.86 (m, 2H, H-8), 6.83 (ddd, ^3J = 8.4 Hz, ^3J = 5.5 Hz, ^5J = 0.4 Hz, 2H, H-3), 6.14 (dd, ^3J = 5.5 Hz, ^4J = 0.8 Hz, 2H, H-2), 3.88 (s, 2H, CH_2) ppm.

HRMS (ESI-TOF, positive mode, $CHCl_3$/MeCN 1:1): *m/z* calculated for $C_{48}H_{31}N_5NaO_8Ru$ $[M+Na]^+$: 930.1108; found 930.1099.

Single Crystal X-Ray analysis

Table S1. Crystal data and structure refinement for compounds **Ru(bda)(Carb-COOMe)** and **Ru(bda)(Carb-COOH)**.

Compound	**Ru(bda)(Carb-COOMe)**	**Ru(bda)(Carb-COOH)**
CCDC Number	2481884	2481885
Empirical Formula	$C_{50.82} H_{40.05} Cl_{1.22} N_5 O_{9.71} Ru$	$C_{49.76}H_{34.52}Cl_{3.52}N_5O_8Ru$
M / g mol^{-1}	1020.45	1056.27
Temperature / K	100(2)	100(2)
Wavelength / Å	1.54178	1.54178
Crystal system, space group	Monoclinic, *P*2(1)	Monoclinic, *P*2(1)/c
Unit cell dimensions:		
a / Å	9.0473(8)	15.7198(17)
b / Å	19.734(3)	16.6062(17)
c / Å	25.434(3)	17.8743(19) A
α / °	90	90
β / °	97.260(7)	96.744(6)
γ / °	90	90
Volume V / Å^3	4504.4(9)	4633.7(8)
Z	4	4
Calculated density ρ_{cal} / g cm^{-3}	1.505	1.514
Absorption coefficient / mm^{-1}	4.043	5.115
F(000)	2089.6	2144
Crystal size / mm^3	0.067 x 0.064 x 0.013	0.128 x 0.122 x 0.110
Measurement range of Θ / °	2.843 to 72.639	2.830 to 72.220
Limiting indices<br>Reflections collected / unique<br>Completeness / %	−11<=h<=10, −22<=k<=24, −31<=l<=31<br>47880 / 16906 [*R*(int) = 0.0412]<br>100.0 %	−19<=h<=19, −19<=k<=20, −2<=l<=22<br>102737 / 9127 [*R*(int) = 0.0351]<br>100.0 %
Absorption correction	Semi-empirical from equivalents	Semi-empirical from equivalents
Max. & Min. transmission	0.7536 and 0.6727	0.7536 and 0.6765
Refinement method	Full-matrix least-squares on F^2	Full-matrix least-squares on F^2
Data / restraints / parameters	16906 / 250 / 1360	9127 / 63 / 643
Goodness of fit for F^2	1.029	1.069
Final *R* indices [$I > 2\sigma(I)$]	*R*1 = 0.0291, *wR*2 = 0.0642	*R*1 = 0.0321, *wR*2 = 0.0856
R indices (all data)	*R*1 = 0.0315, *wR*2 = 0.0653	*R*1 = 0.0355, *wR*2 = 0.0890
Largest diff. peak and hole	0.559 and –0.357 e.A^3	1.623 and –0.525 e.A^3

Photocatalytic Water Oxidation

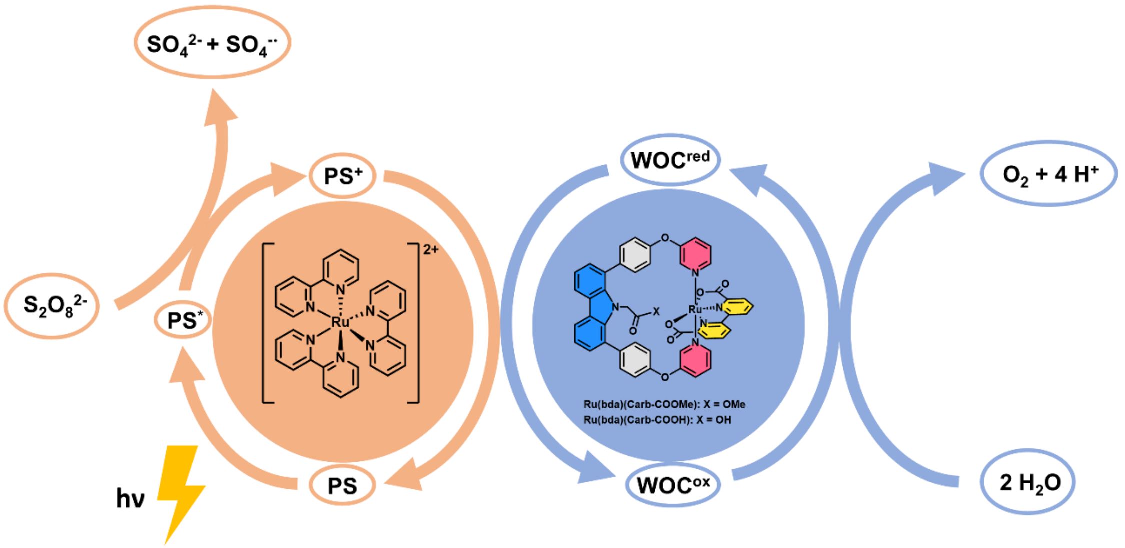

Figure S1. Schematic presentation of the photocatalytic water oxidation cycle in a three-component system containing $Na_2S_2O_8$ as sacrificial electron acceptor (SAE), $[Ru(bpy)_3]^{2+}$ as photosensitizer (PS) and complexes **Ru(bda)(Carb-COOMe)** and **Ru(bda)(Carb-COOH)** as water oxidation catalyst (WOC).

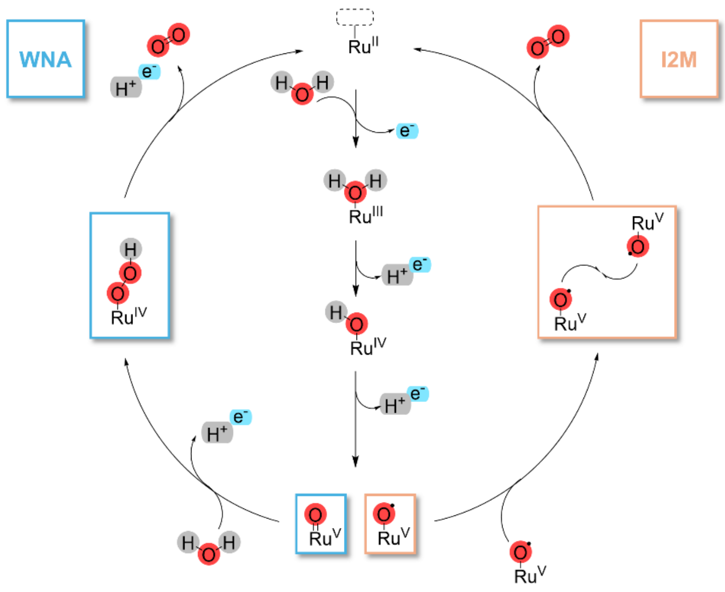

Figure S2. Mechanistic scheme representing the two possible pathways for homogeneous water oxidation: single-site water nucleophilic attack (**WNA**) and bimolecular interaction of two metal-oxo species (**I2M**).

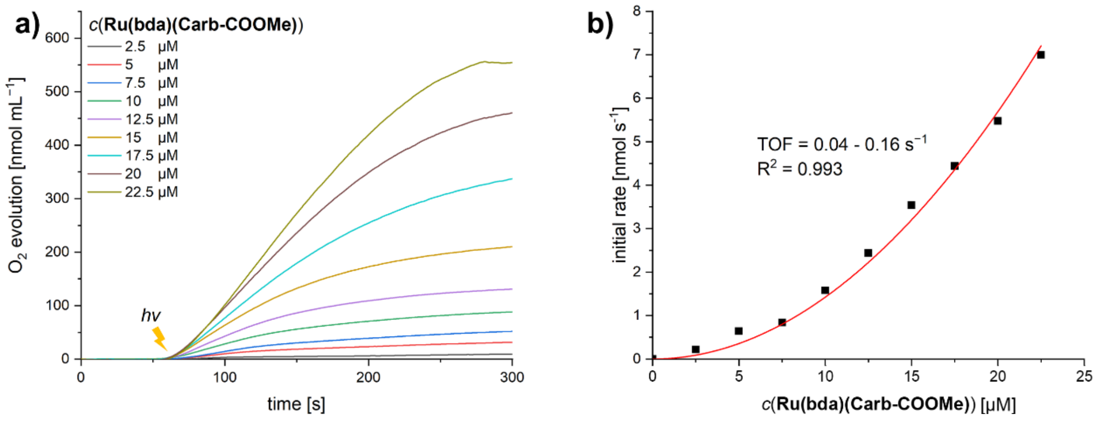

Figure S3. a) Photocatalytic water oxidation experiments with **Ru(bda)(Carb-COOMe)** as WOC in CH_3CN/H_2O 4:6 (**pH 7**, 50 mM phosphate buffer), *c*(PS) = 1.5 mM, *c*($Na_2S_2O_8$) = 37 mM. The lightning symbol indicates the start of sample irradiation at *t* = 50 s. **b**) Plot of the initial rates vs. the catalyst concentration with quadratic regression.

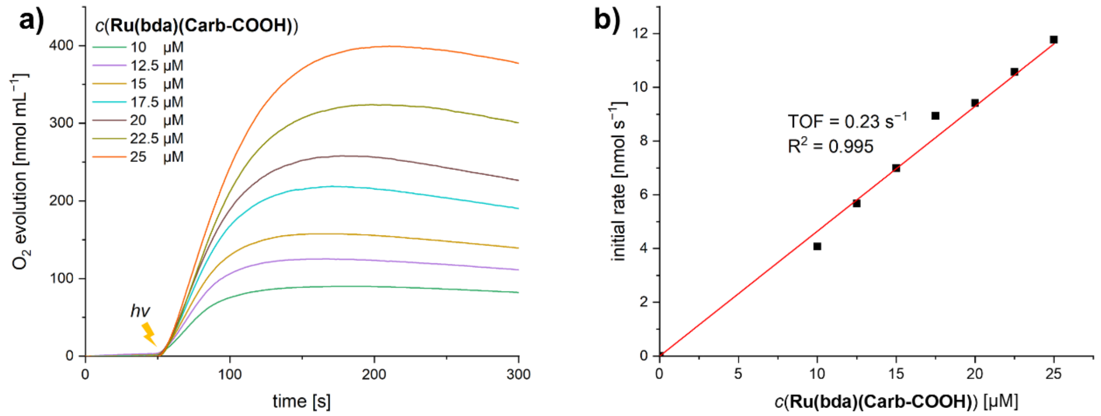

Figure S4. a) Photocatalytic water oxidation experiments with **Ru(bda)(Carb-COOH)** as WOC in CH_3CN/H_2O 4:6 (**pH 7**, 50 mM phosphate buffer), *c*(PS) = 1.5 mM, *c*($Na_2S_2O_8$) = 37 mM. The lightning symbol indicates the start of sample irradiation at *t* = 50 s. **b**) Plot of the initial rates vs. the catalyst concentration with linear regression.

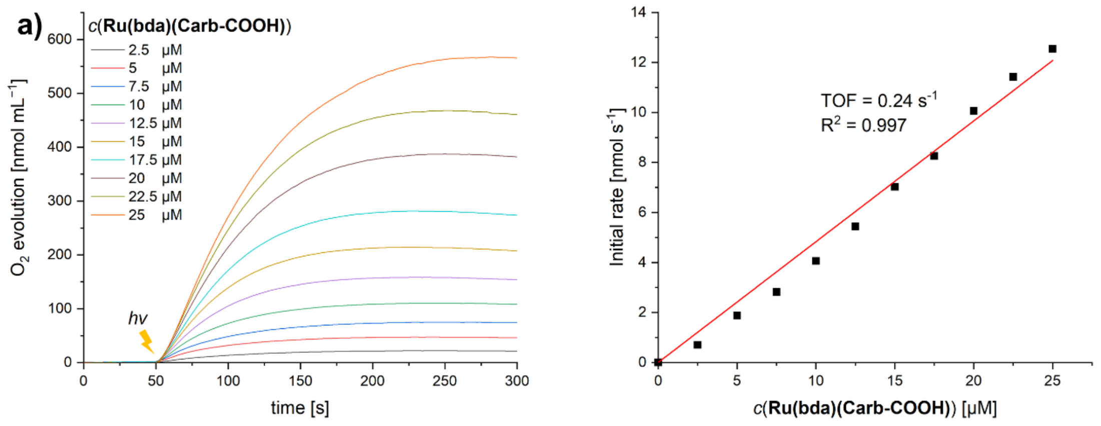

Figure S5. a) Photocatalytic water oxidation experiments with **Ru(bda)(Carb-COOH)** as WOC in CH_3CN/H_2O 4:6 (**pH 9**, 50 mM phosphate buffer), *c*(PS) = 1.5 mM, *c*($Na_2S_2O_8$) = 37 mM. The lightning symbol indicates the start of sample irradiation at *t* = 50 s. **b**) Plot of the initial rates vs. the catalyst concentration with linear regression.

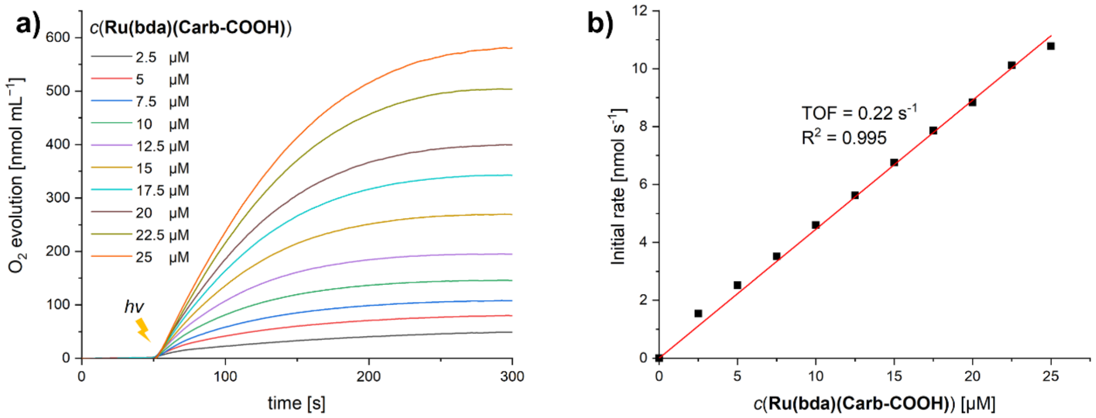

Figure S6. a) Photocatalytic water oxidation experiments with **Ru(bda)(Carb-COOH)** as WOC in CH_3CN/H_2O 4:6 (**pH 10**, 50 mM phosphate buffer), *c*(PS) = 1.5 mM, *c*($Na_2S_2O_8$) = 37 mM. The lightning symbol indicates the start of sample irradiation at *t* = 50 s. **b**) Plot of the initial rates vs. the catalyst concentration with linear regression.

Table S2. Catalytic Activities and proposed water oxidation mechanisms for selected mononuclear Ru(bda)-based WOCs under photocatalytic conditions.

Catalyst	TOF_{max} [s^{-1}]	TON	Water Oxidation Mechanism
**Ru(bda)(Carb-COOH)**	0.23	16	Oxide relay
**Ru(bda)(Carb-COOMe)**	0.16	25	I2M
**6**[30,31]	0.35	10	I2M
**7**[32,33]	0.24	140	I2M
**M1**[34]	0.10	10	I2M
**M2**[34]	3.2	150	I2M
**8**[35]	0.12	9	I2M
**9**[35]	0.50	27	WNA
**10**[35]	0.22	25	WNA

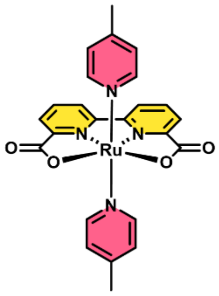

6

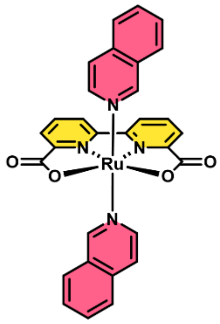

7

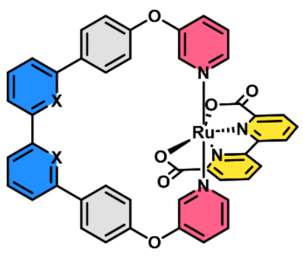

M1: X = N
M2: X = C-H

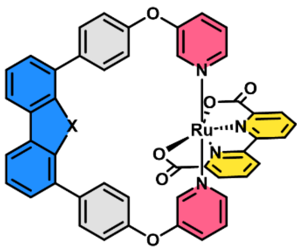

8: X = O
9: X = S
10: X = N-H

Kinetic Isotope Effect

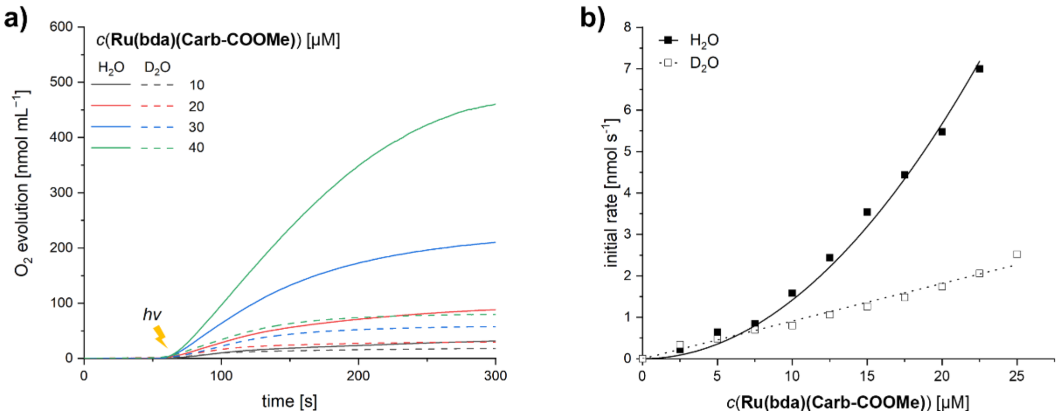

Figure S7. a) Photocatalytic water oxidation experiments with **Ru(bda)(Carb-COOMe)** as WOC in CH_3CN/H_2O or CH_3CN/D_2O 4:6 (pH 7, 50 mM phosphate buffer), *c*(PS) = 1.5 mM, *c*($Na_2S_2O_8$) = 37 mM. The lighting symbol indicates the start of sample irradiation at *t* = 50 s. **b**) Plot of the initial rates vs. the catalyst concentration.

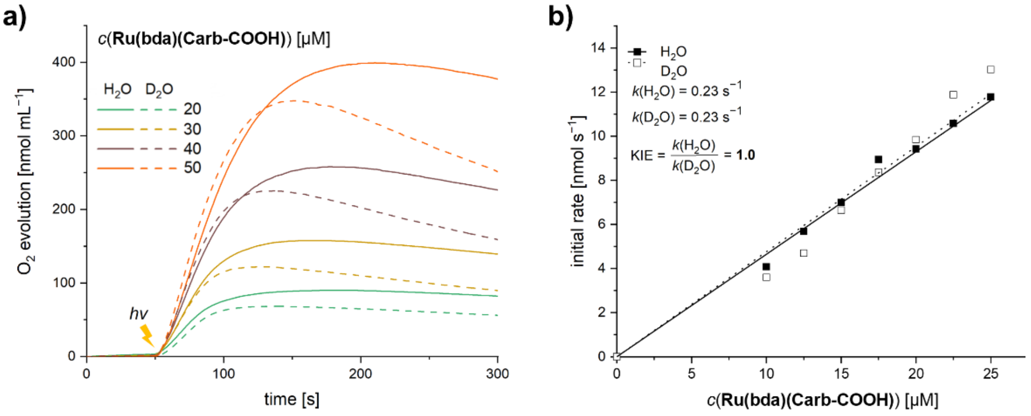

Figure S8. a) Photocatalytic water oxidation experiments with **Ru(bda)(Carb-COOH)** as WOC in CH_3CN/H_2O or CH_3CN/D_2O 4:6 (pH 7, 50 mM phosphate buffer), *c*(PS) = 1.5 mM, *c*($Na_2S_2O_8$) = 37 mM. The lighting symbol indicates the start of sample irradiation at *t* = 50 s. **b**) Plot of the initial rates vs. the catalyst concentration with linear regression for the determination of the individual reaction rates *k*(H_2O) and *k*(D_2O).

Electrochemistry

Table S3. Redox properties of the complexes **Ru(bda)(Carb-COOMe)** and **Ru(bda)(Carb-COOH)** under neutral (pH 7) aqueous conditions with 50% 2,2,2-trifluoroethanol (TFE) as organic cosolvent. The measurements were performed in 50 mM ionic strength phosphate buffered aqueous mixtures at $c = 2.5 \cdot 10^{-4}$ M.

**Conditions**	**1:1 TFE/H_2O (pH 7)**		
**Catalyst**	*E vs.* NHE [V]		
	Ru^{III}/Ru^{II}	Ru^{IV}/Ru^{III}	Ru^{V}/Ru^{IV}
**Ru(bda)(Carb-COOMe)**	+ 0.62	+0.85	+ 1.01
**Ru(bda)(Carb-COOH)**	+ 0.68	+ 0.86	+ 1.01

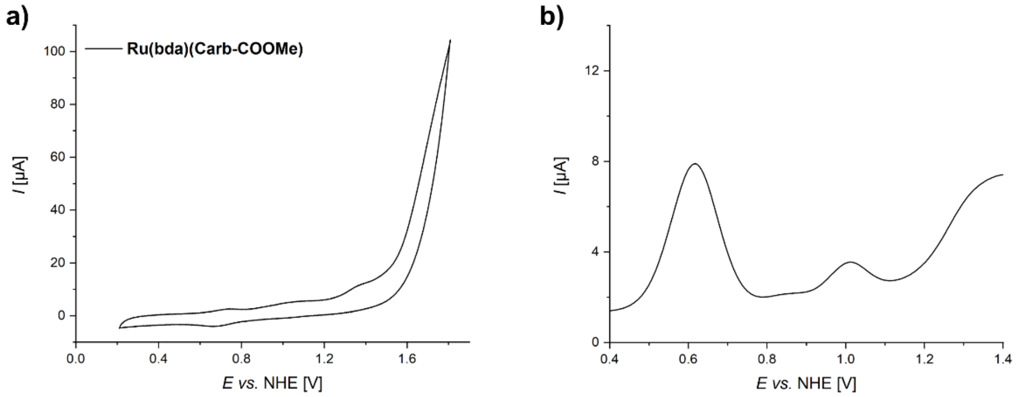

Figure S9. a) CV and **b)** DPV of **Ru(bda)(Carb-COOMe)** in TFE/H_2O 1:1 (pH 7, phosphate buffer, $c = 2.5{\cdot}10^{-4}$ M).

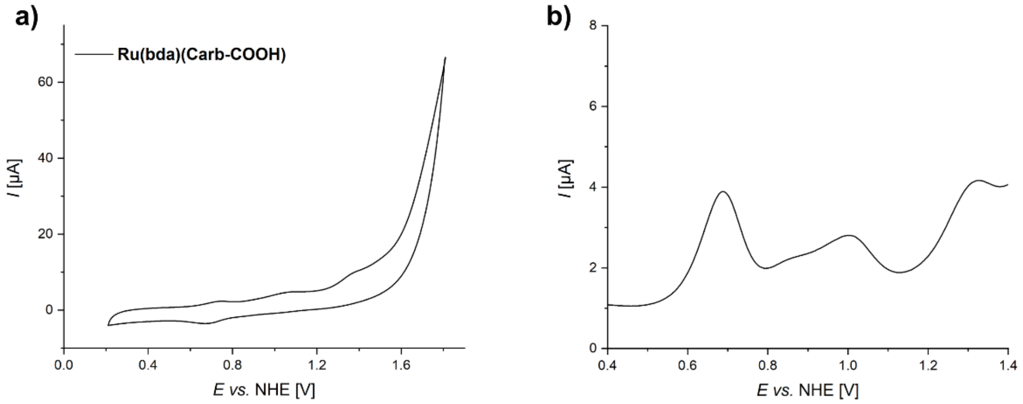

Figure S10. a) CV and **b)** DPV of **Ru(bda)(Carb-COOH)** in TFE/H_2O 1:1 (pH 7, phosphate buffer, $c = 2.5{\cdot}10^{-4}$ M).

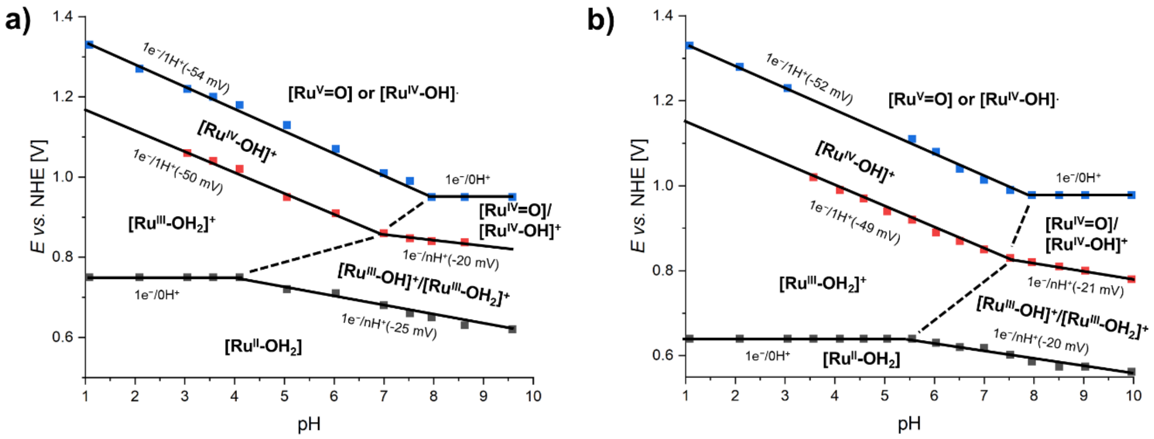

Figure S11. Pourbaix diagrams of a) **Ru(bda)(Carb-COOH)** and b) **Ru(bda)(Carb-COOMe)**. Differential pulse voltammetry measurements were conducted in a 1:1 H_2O/TFE mixture (phosphate buffer at different pH values with *I* = 0.1 M, *c*(**WOC**) = $2.5 \cdot 10^{-4}$ M). For both compounds the Ru^{III}/Ru^{IV} redox process could not be reliably resolved over the entire pH range, as the corresponding oxidation peak exhibited only weak electrochemical signals (see Figure S9b and S10b). However, missing data points in the respective pH ranges could be rationally extrapolated, as shown in the diagram.

VT-NMR spectra

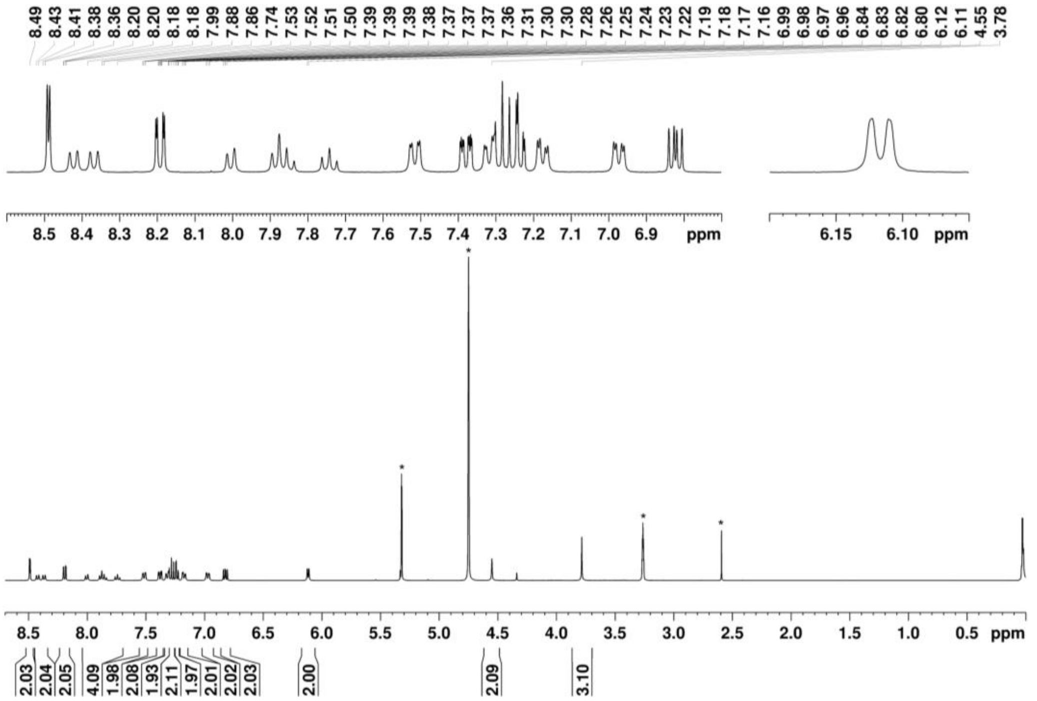

Figure S12. ^{1}H NMR spectrum (400 MHz, CD_2Cl_2/ MeOD 4:1, 252 K) of **Ru(bda)(Carb-COOMe)** (* residual solvent).

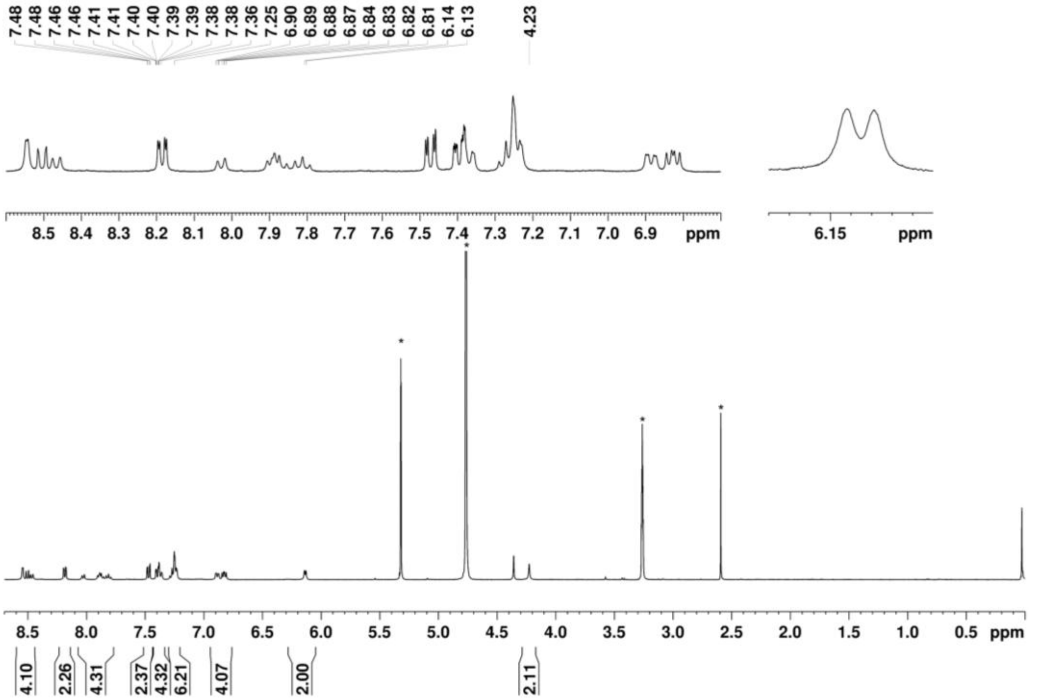

Figure S13. ^{1}H NMR spectrum (400 MHz, CD_2Cl_2/ MeOD 4:1, 252 K) of **Ru(bda)(Carb-COOH)** (* residual solvent).

NMR spectra

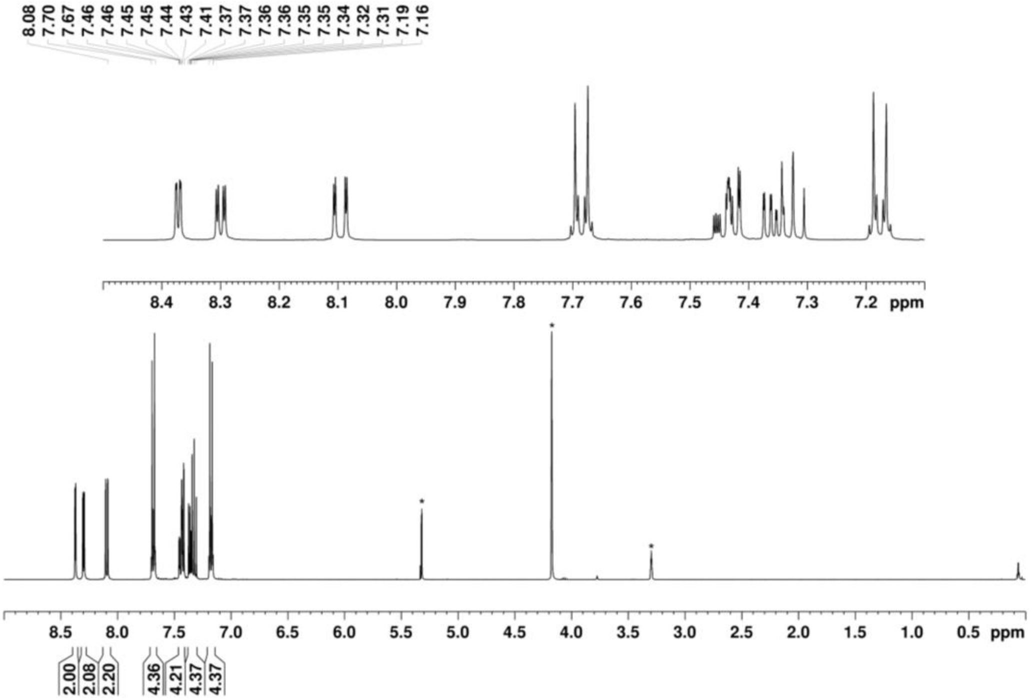

Figure S14. ^{1}H NMR spectrum (400 MHz, CD_2Cl_2/ MeOD 4:1, 295 K) of **3** (* residual solvent).

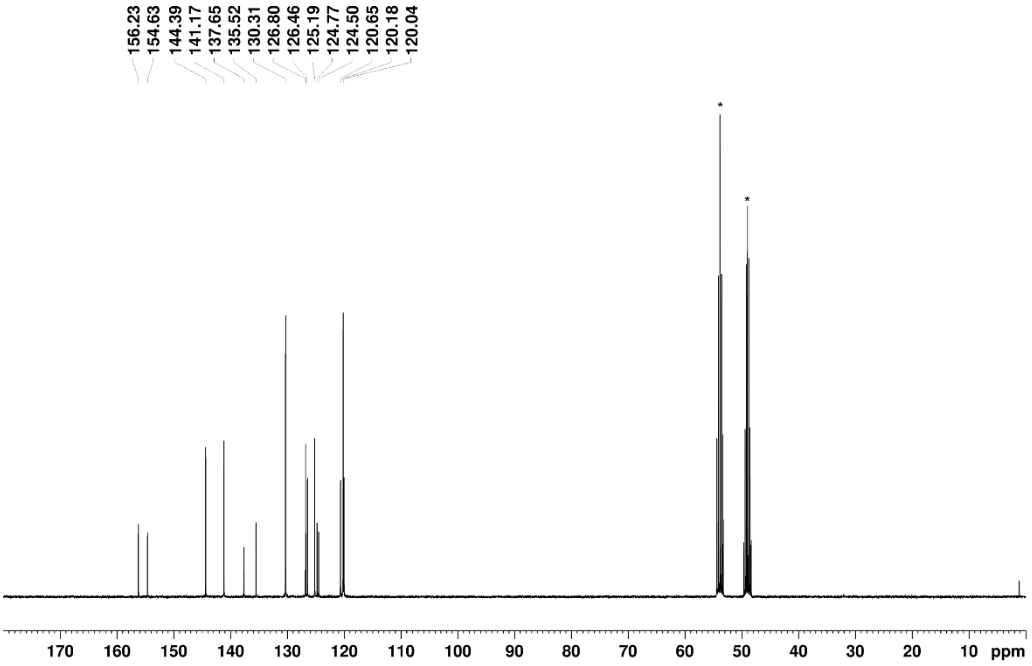

Figure S15. ^{13}C NMR spectrum (100 MHz, CD_2Cl_2/ MeOD 4:1, 295 K) of **3** (* residual solvent).

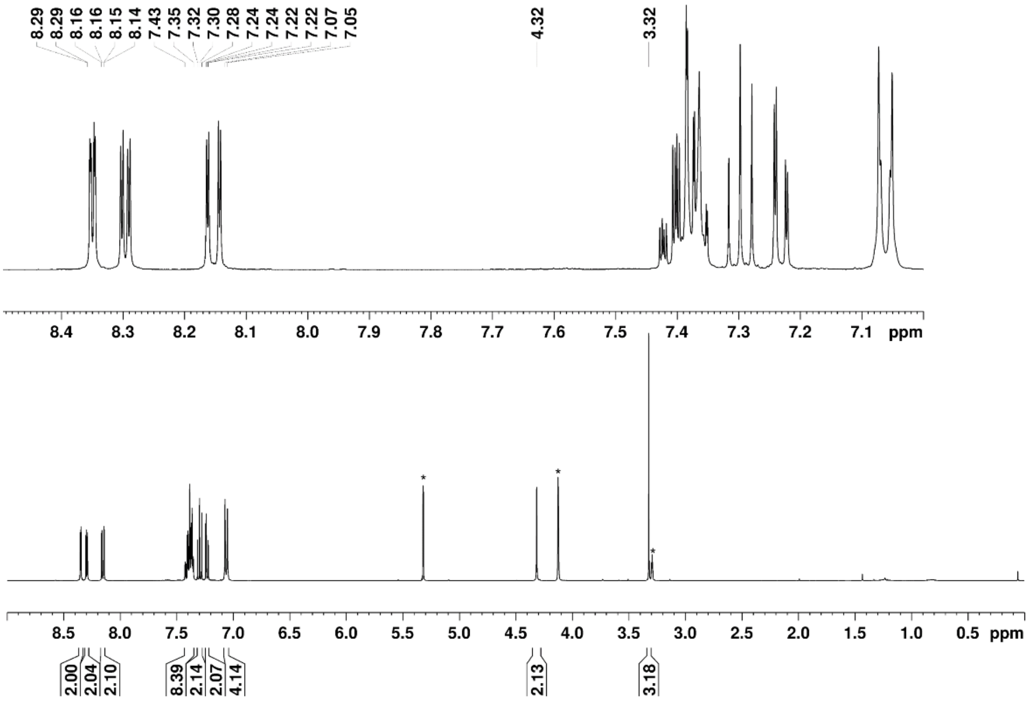

Figure S16. ^{1}H NMR spectrum (400 MHz, CD_2Cl_2/ MeOD 4:1, 295 K) of **4** (* residual solvent).

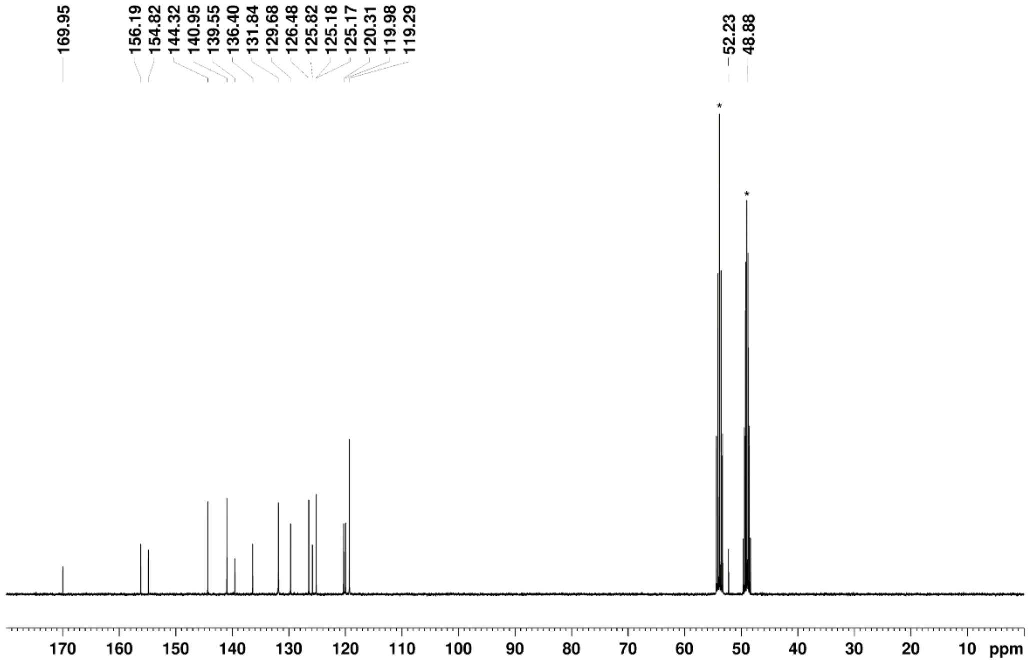

Figure S17. ^{13}C NMR spectrum (100 MHz, CD_2Cl_2/ MeOD 4:1, 295 K) of **4** (* residual solvent).

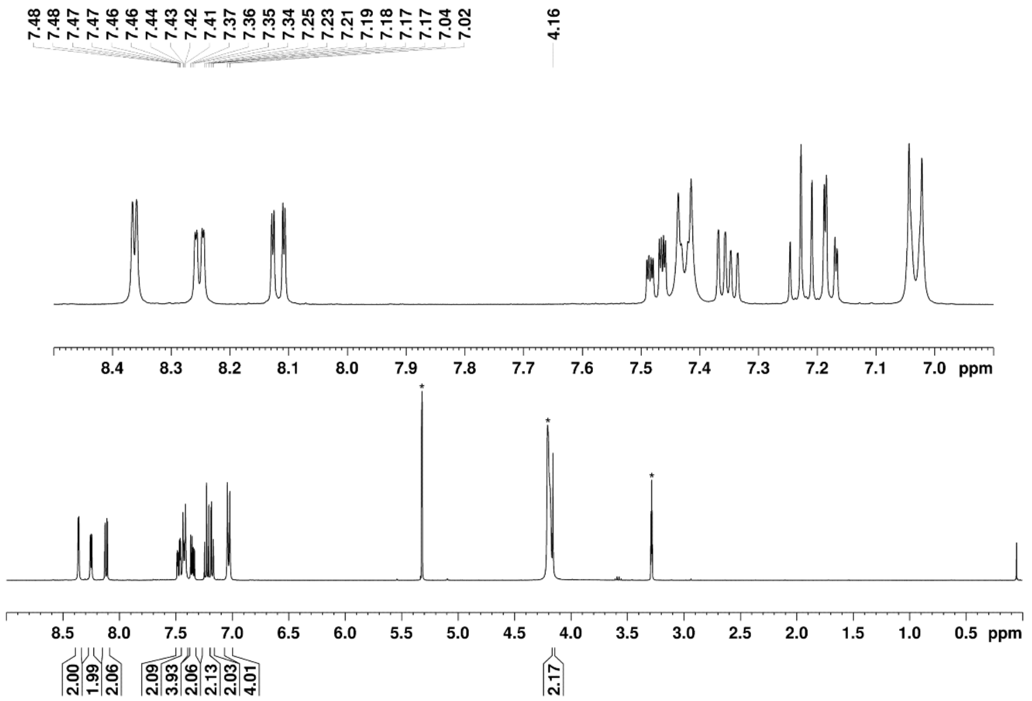

Figure S18. ^{1}H NMR spectrum (400 MHz, CD_2Cl_2/ MeOD 4:1, 295 K) of **5** (* residual solvent).

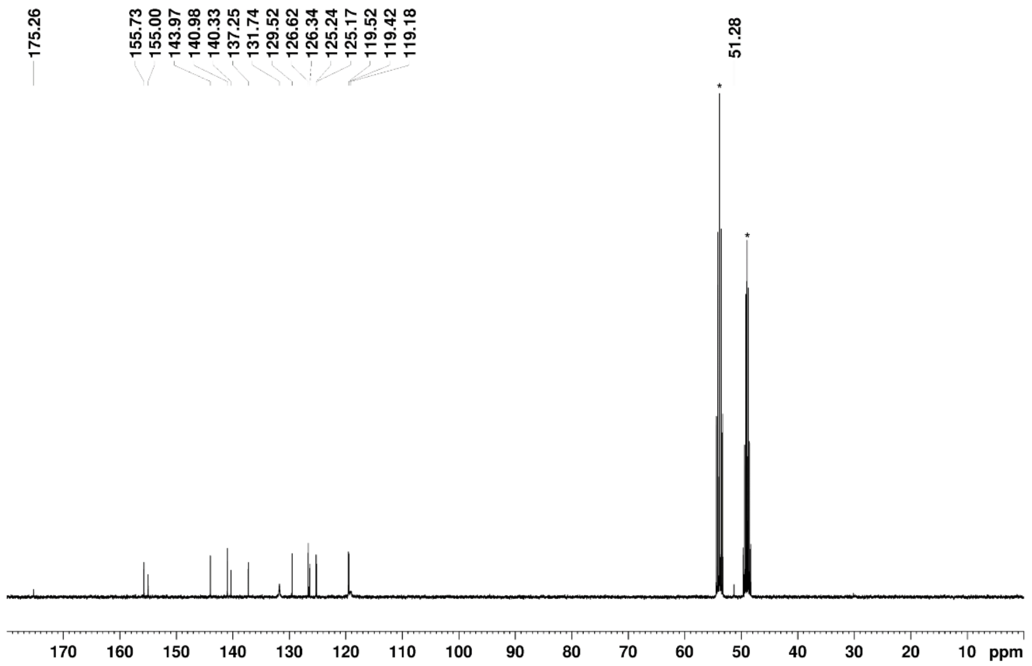

Figure S19. ^{13}C NMR spectrum (100 MHz, CD_2Cl_2/ MeOD 4:1, 295 K) of **5** (* residual solvent).

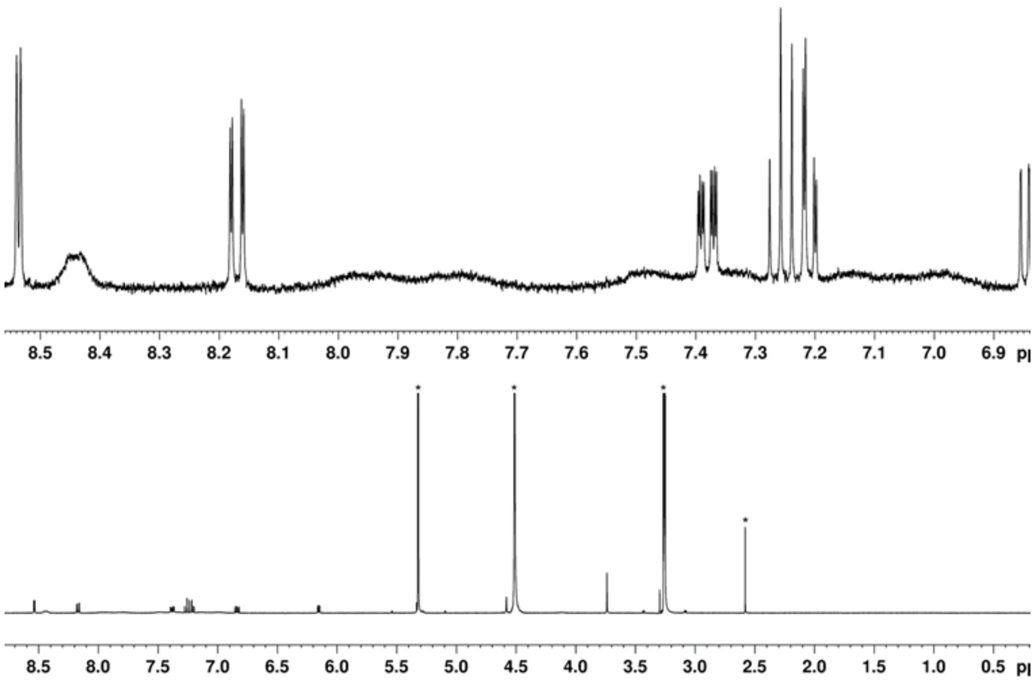

Figure S20. ^{1}H NMR spectrum (400 MHz, CD_2Cl_2/ MeOD 4:1, 295 K) of **Ru(bda)(Carb-COOMe)** (* residual solvent). Severe line broadening in the aromatic region of the spectrum is clearly visible.

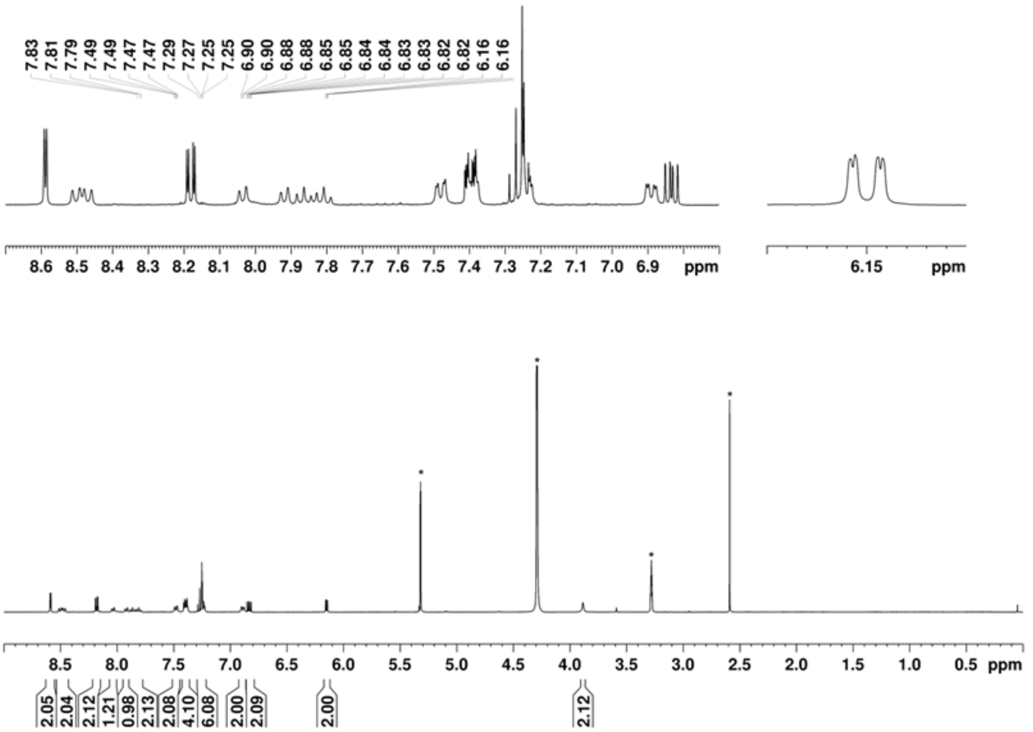

Figure S21. ^{1}H NMR spectrum (400 MHz, CD_2Cl_2/ MeOD 4:1, 295 K) of **Ru(bda)(Carb-COOH)** (* residual solvent).

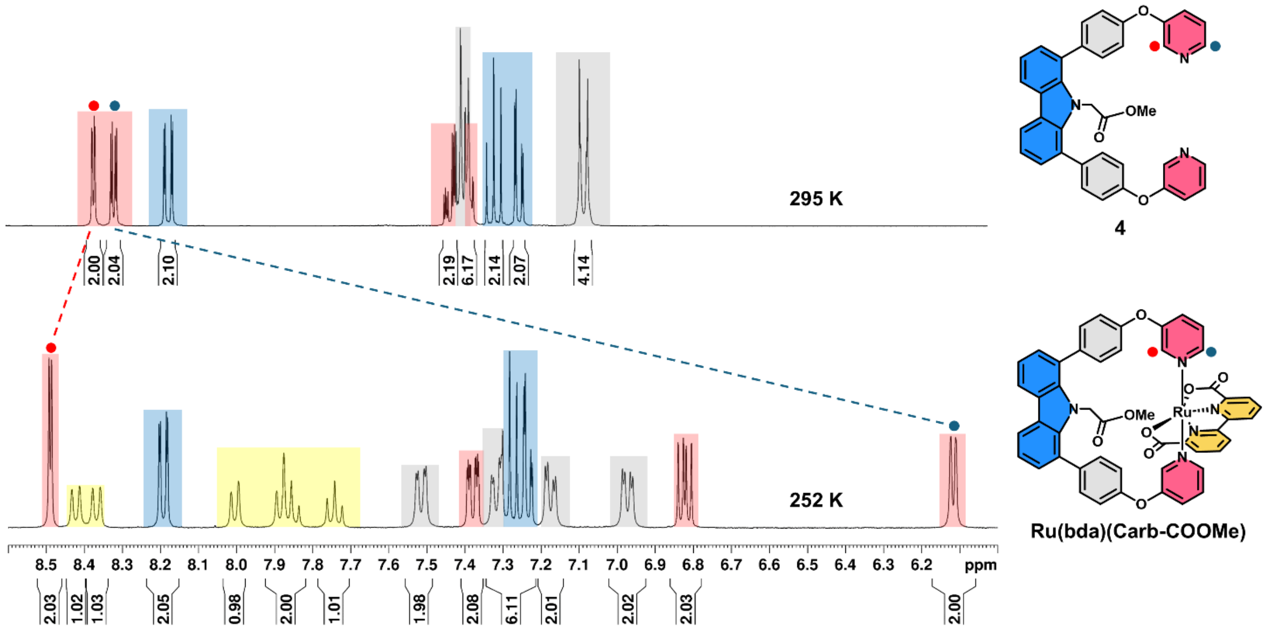

Figure S22. ^{1}H NMR spectra (4:1 CD_2Cl_2/MeOD, 400 MHz, variable temperatures) of **4** and **Ru(bda)(Carb-COOMe)** with colour coded signals according to the molecular structures on the right. The colours correspond to bda (yellow), axial pyridine units (red), phenylene group (grey) and carbazole moiety (blue).

HR mass spectra

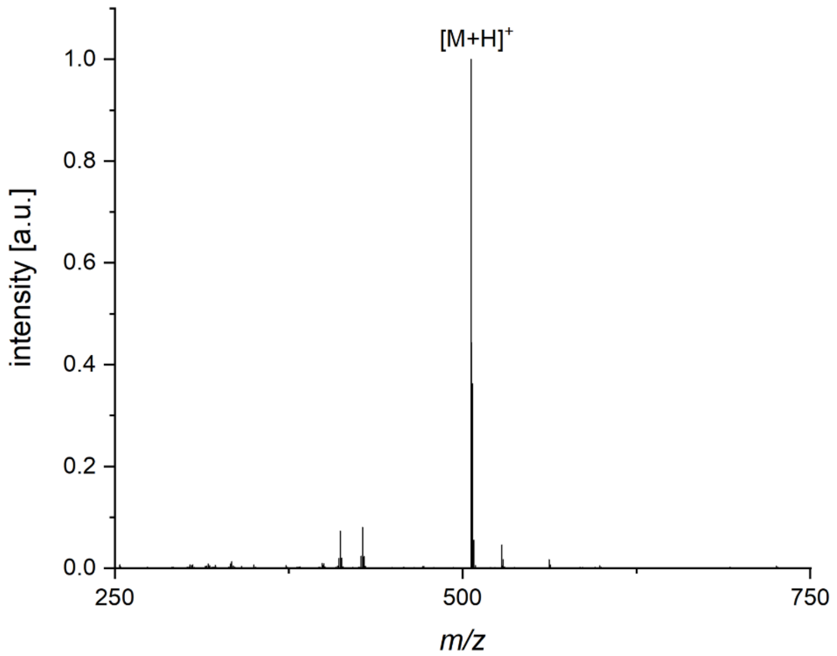

Figure S23. HRMS (ESI-TOF) mass spectrum ($CH_3CN/CHCl_3$ 1:1, positive mode) of **3**.

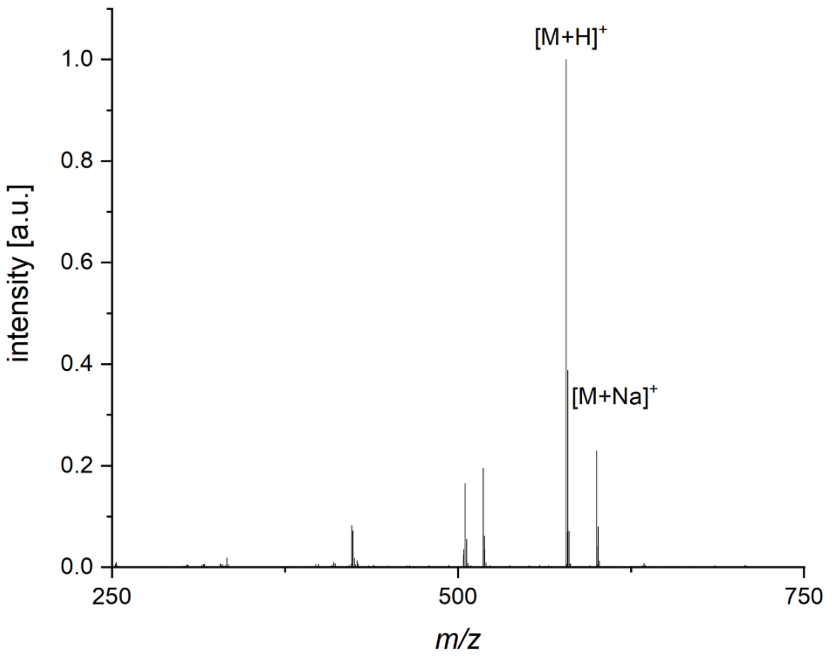

Figure S24. HRMS (ESI-TOF) mass spectrum ($CH_3CN/CHCl_3$ 1:1, positive mode) of **4**.

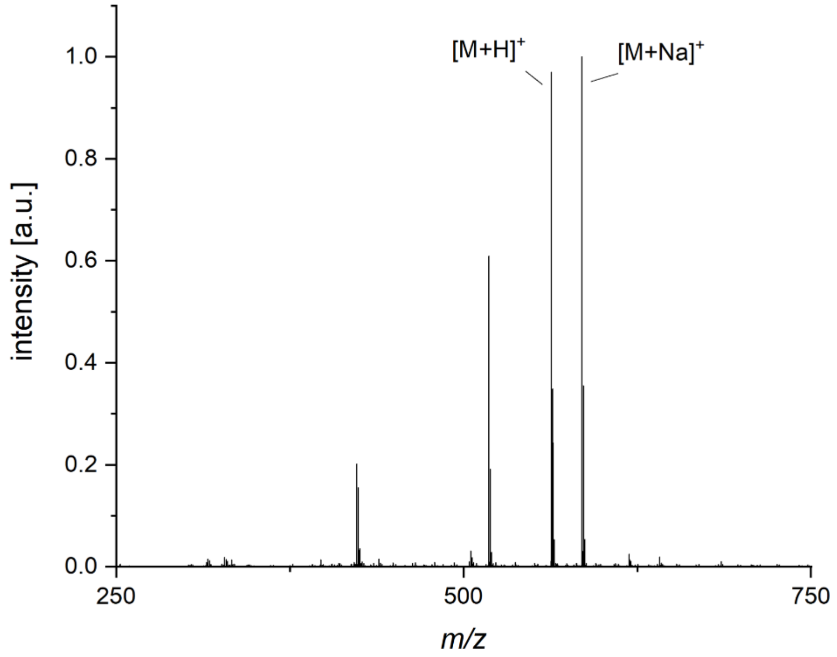

Figure S25. HRMS (ESI-TOF) mass spectrum ($CH_3CN/CHCl_3$ 1:1, positive mode) of **5**.

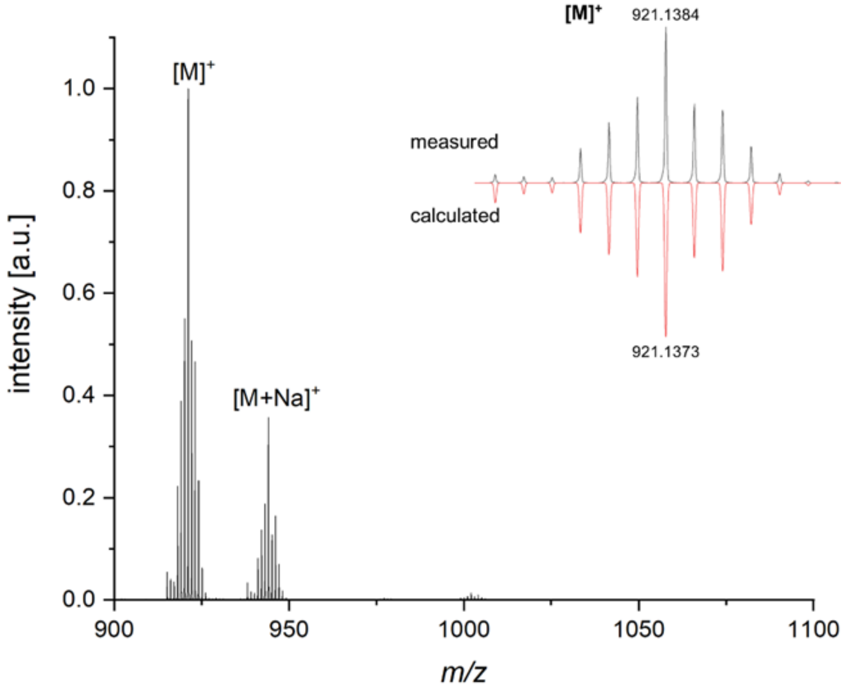

Figure S26. HRMS (ESI-TOF) mass spectrum ($CH_3CN/CHCl_3$ 1:1, positive mode) of **Ru(bda)(Carb-COOMe)** with the inset showing the measured and calculated isotopic distribution of $[M+Na]^+$.

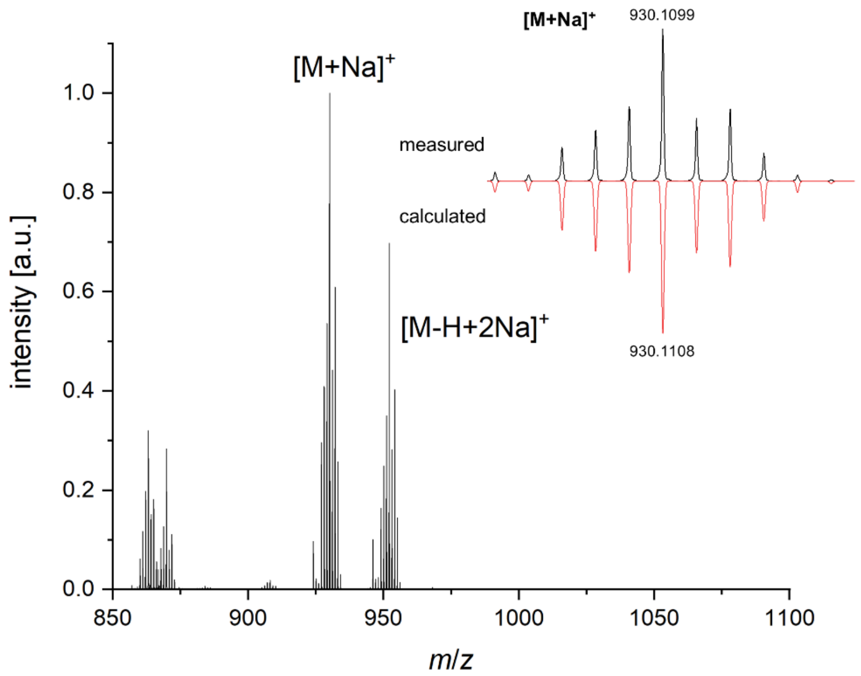

Figure S27. HRMS (ESI-TOF) mass spectrum ($CH_3CN/CHCl_3$ 1:1, positive mode) of **Ru(bda)(Carb-COOH)** with the inset showing the measured and calculated isotopic distribution of $[M]^+$.

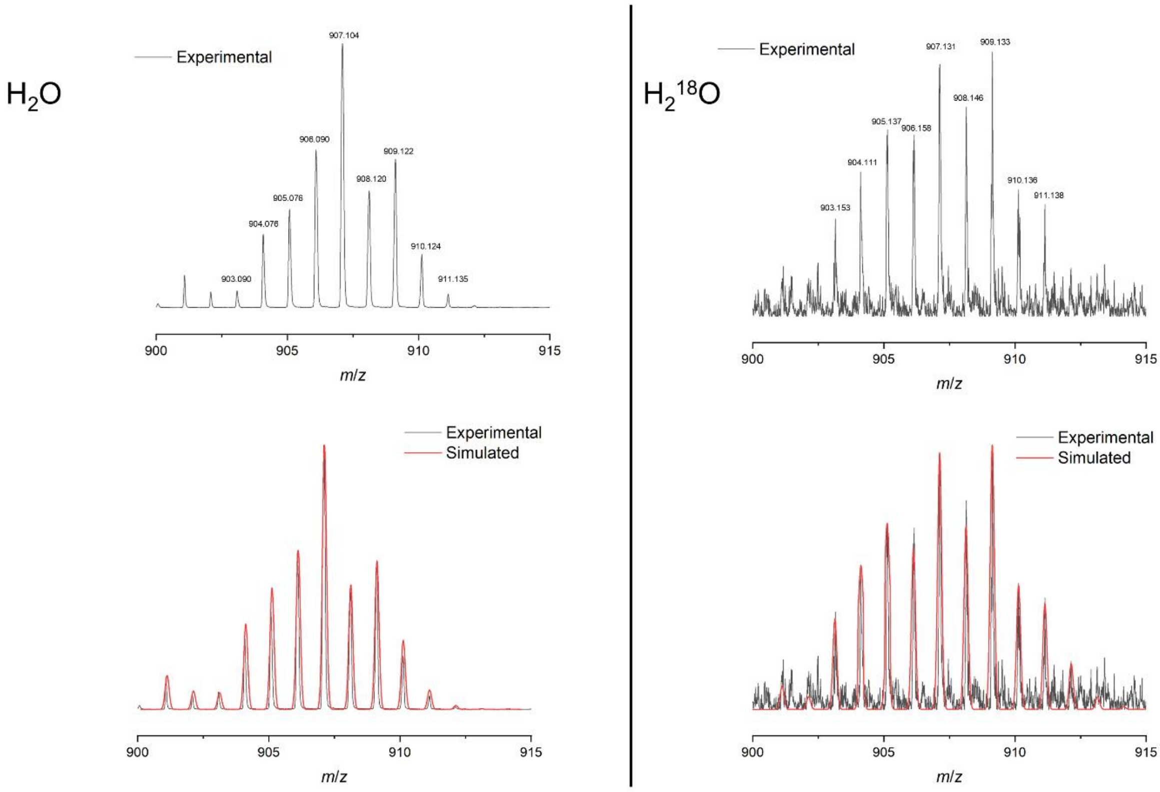

Figure S28. HR MALDI mass spectrum (positive mode, DCTB) of post-photocatalytic samples of **Ru(bda)(Carb-COOH)** using unlabeled (left) and ^{18}O-labeled (right) water. The isotope pattern for the ^{18}O-labeled sample corresponds to a ratio of of [**Ru(bda)(Carb-COOH)**]$^+$: [**Ru(bda)(Carb-CO^{18}OH)**]$^+$ = 1:1.

Theoretical Calculations

The energetics of the oxide relay mechanism was investigated by calculating the electronic energy profile for the O-O approaching (step 1) and H_2O attacking (step 2) steps. As shown in Figure S29a, the electronic energy barrier for the O-O approaching step has a quite low value of 76.1 kJ mol^{-1}. The H_2O attacking step shows an electronic energy barrier of 84.2 kJ mol^{-1}, as shown in Figure S29b. All these calculations were done with a low spin state (spin multiplicity 2), as it is generally recognized as the stable spin state for Ru(bda) catalysts.[26-28] This was also confirmed by calculations using a high spin state (spin multiplicity 4).

While these calculations offer valuable insights, they are not intended to provide an exhaustive and very accurate description of the activation barriers. More sophisticated wavefunction-based methods as well as sampling methods, such as ab initio/DFT-based metadynamics with explicit solvation, could yield a more realistic energetic landscape, as demonstrated in our previous works.[20,36-39]

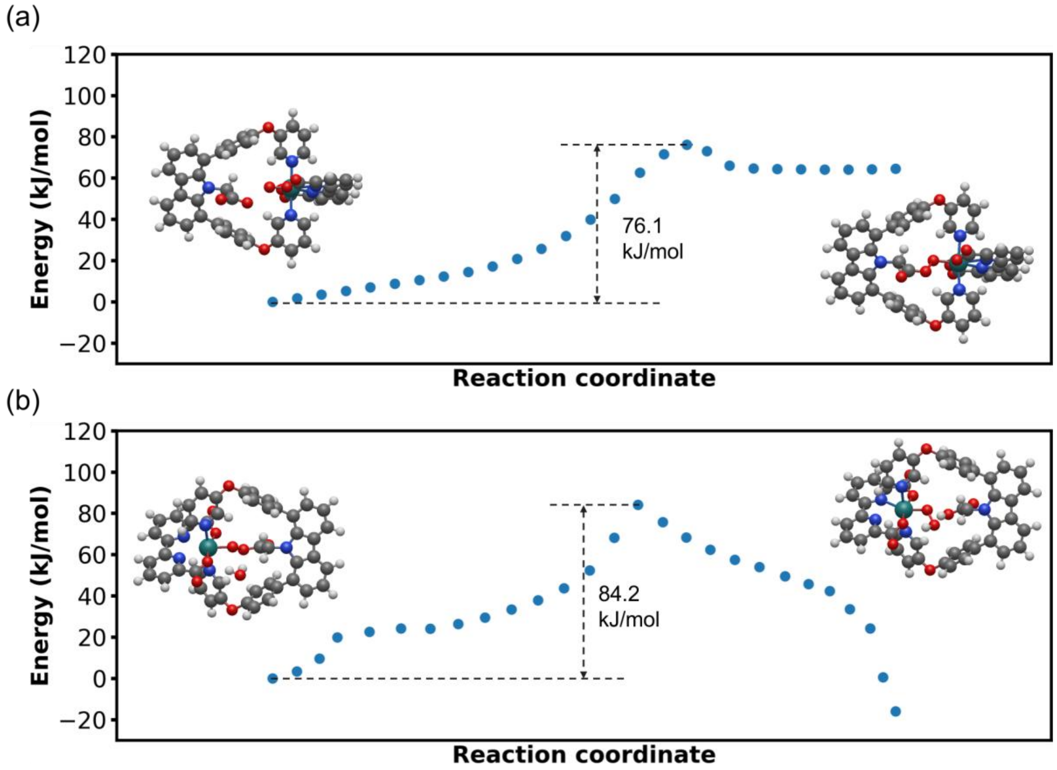

Figure S29. Calculated electronic energy profile of the oxide relay mechanism for **Ru(bda)(Carb-COOH)**: (a) step 1 (O-O approaching), (b) step 2 (H_2O attacking).

References

[1] C. L. Donnici, D. H. Máximo Filho, L. L. C. Moreira, G. T. d. Reis, E. S. Cordeiro, I. M. F. d. Oliveira, S. Carvalho, E. B. Paniago, "Synthesis of the Novel 4,4'-and 6,6'-Dihydroxamic-2,2'-Bipyridines and Improved Routes to 4,4'-and 6,6'-Substituted 2,2'-Bipyridines and Mono-N-Oxide-2,2'-Bipyridine" *J. Braz. Chem. Soc.* **1998**, *9*, 455-460.

[2] I. Evans, A. Spencer, G. Wilkinson, "Dichlorotetrakis (Dimethyl Sulphoxide) Ruthenium (II) and its Use as a Source Material for Some New Ruthenium (II) Complexes" *J. Chem. Soc., Dalton Trans.* **1973**, 204-209.

[3] E. Dulière, M. Devillers, J. Marchand-Brynaert, "Novel Phosphinite−Ruthenium (II) Complexes Covalently Bound on Silica: Synthesis, Characterization, and Catalytic Behavior versus Oxidation Reactions of Alcohols into Aldehydes" *Organometallics* **2003**, *22*, 804-811.

[4] F. Li, B. Zhang, X. Li, Y. Jiang, L. Chen, Y. Li, L. Sun, "Highly Efficient Oxidation of Water by a Molecular Catalyst Immobilized on Carbon Nanotubes" *Angew. Chem. Int. Ed.* **2011**, *50*, 12276-12279.

[5] S. J. Malthus, S. A. Cameron, S. Brooker, "Improved access to 1,8-Diformyl-Carbazoles Leads to Metal-free Carbazole-based [2+2] Schiff Base Macrocycles With Strong Turn-On Fluorescence Sensing of Zinc (II) Ions" *Inorg. Chem.* **2018**, *57*, 2480-2488.

[6] J. M. Cid, G. Duvey, G. Tresadern, V. Nhem, R. Furnari, P. Cluzeau, J. A. Vega, A. I. de Lucas, E. Matesanz, J. M. Alonso, M. L. Linares, J. I. Andrés, S. M. Poli, R. Lutjens, H. Himogai, J. P. Rocher, G. J. Macdonald, D. Oehlrich, H. Lavreysen, A. Ahnaou, W. Drinkenburg, C. Mackie, A. A. Trabanco, "Discovery of 1,4-Disubstituted 3-Cyano-2-Pyridones: a New Class of Positive Allosteric Modulators of the Metabotropic Glutamate 2 Receptor" *J. Med. Chem.* **2012**, *55*, 2388-2405.

[7] G. R. Fulmer, A. J. Miller, N. H. Sherden, H. E. Gottlieb, A. Nudelman, B. M. Stoltz, J. E. Bercaw, K. I. Goldberg, "NMR Chemical Shifts of Trace Impurities: Common Laboratory Solvents, Organics, and Gases in Deuterated Solvents Relevant to the Organometallic Chemist" *Organometallics* **2010**, *29*, 2176-2179.

[8] S. Gaweda, G. Stochel, K. Szaciłowski, "Photosensitization and Photocurrent Switching in Carminic Acid/Titanium Dioxide Hybrid Material" *J. Phys. Chem. C* **2008**, *112*, 19131-19141.

[9] G. M. Sheldrick, "Crystal Structure Refinement With SHELXL" *Acta Crystallogr., Sect. C:Cryst. Struct. Commun.* **2015**, *71*, 3-8.

[10] G. M. Sheldrick, "A Short History of SHELX" *Acta Crystallogr., Sect. A:Found. Crystallogr.* **2008**, *64*, 112-122.

[11] A. L. Spek, “PLATON SQUEEZE: A Tool for the Calculation of the Disordered Solvent Contribution to the Calculated Structure Factors” *Acta Crystallogr., Sect. C:Cryst. Struct. Commun.* **2015**, *71*, 9-18.

[12] A. L. Spek, “Structure Validation in Chemical Crystallography” *Acta Crystallogr., Sect. D:Struct. Biol.* **2009**, *65*, 148-155.

[13] G. Henkelman, B. P. Uberuaga, H. Jónsson, “A Climbing Image Nudged Elastic Band Method for Finding Saddle Points and Minimum Energy Paths” *J. Chem. Phys.* **2000**, *113*, 9901-9904.

[14] F. Neese, “The ORCA Program System” *Wiley Interdiscip. Rev. Comput. Mol. Sci.* **2012**, *2*, 73-78.

[15] F. Neese, “Software Update: The ORCA Program System—Version 6.0” *Wiley Interdiscip. Rev. Comput. Mol. Sci.* **2025**, *15*, e70019.

[16] F. Weigend, R. Ahlrichs, “Balanced Basis Sets of Split Valence, Triple Zeta Valence and Quadruple Zeta Valence Quality for H to Rn: Design and Assessment of Accuracy” *Phys. Chem. Chem. Phys.* **2005**, *7*, 3297-3305.

[17] A. D. Becke, “Density-Functional Exchange-Energy Approximation With Correct Asymptotic Behavior” *Phys. Rev. A* **1988**, *38*, 3098-3100.

[18] C. Lee, W. Yang, R. G. Parr, “Development of the Colle-Salvetti Correlation-Energy Formula into a Functional of the Electron Density” *Phys. Rev. B* **1988**, *37*, 785-789.

[19] A. D. Becke, “Density-Functional Thermochemistry. III. The Role of Exact Exchange” *The J. Chem. Phys.* **1993**, *98*, 5648-5652.

[20] M. Schilling, M. Böhler, S. Luber, “Towards the Rational Design of the Py5-Ligand Framework for Ruthenium-based Water Oxidation Catalysts” *Dalton Trans.* **2018**, *47*, 10480-10490.

[21] M. Schilling, G. R. Patzke, J. Hutter, S. Luber, “Computational Investigation and Design of Cobalt Aqua Complexes for Homogeneous Water Oxidation” *J. Phys. Chem. C* **2016**, *120*, 7966-7975.

[22] F. H. Hodel, S. Luber, “Redox-Inert Cations Enhancing Water Oxidation Activity: The Crucial Role of Flexibility” *ACS Catal.* **2016**, *6*, 6750-6761.

[23] S. Grimme, J. Antony, S. Ehrlich, H. Krieg, “A Consistent and Accurate Ab Initio Parametrization of Density Functional Dispersion Correction (DFT-D) for the 94 Elements H-Pu” *J. Chem. Phys.* **2010**, *132*.

[24] S. Grimme, S. Ehrlich, L. Goerigk, "Effect of the Damping Function in Dispersion Corrected Density Functional Theory" *J. Comput. Chem.* **2011**, *32*, 1456-1465.

[25] V. Barone, M. Cossi, "Quantum Calculation of Molecular Energies and Energy Gradients in Solution by a Conductor Solvent Model" *J. Phys. Chem.* **1998**, *102*, 1995-2001.

[26] J. A. Stull, T. A. Stich, J. K. Hurst, R. D. Britt, "Electron Paramagnetic Resonance Analysis of a Transient Species Formed During Water Oxidation Catalyzed by the Complex Ion $[(bpy)_2Ru(OH_2)]_2O^{4+}$" *Inorg. Chem.* **2013**, *52*, 4578-4586.

[27] Q. Daniel, P. Huang, T. Fan, Y. Wang, L. Duan, L. Wang, F. Li, Z. Rinkevicius, F. Mamedov, M. S. G. Ahlquist, S. Styring, L. Sun, "Rearranging From 6- to 7-Coordination Initiates the Catalytic Activity: An EPR Study on a Ru-bda Water Oxidation Catalyst" *Coord. Chem. Rev.* **2017**, *346*, 206-215.

[28] G. Li, M. S. G. Ahlquist, "Computational Comparison of Ru(bda)(py)2 and Fe(bda)(py)2 as Water Oxidation Catalysts" *Dalton Trans.* **2022**, *51*, 8618-8624.

[29] V. Ásgeirsson, B. O. Birgisson, R. Bjornsson, U. Becker, F. Neese, C. Riplinger, H. Jónsson, "Nudged Elastic Band Method for Molecular Reactions Using Energy-Weighted Springs Combined with Eigenvector Following" *J. Chem. Theory Comput.* **2021**, *17*, 4929-4945.

[30] L. Wang, L. Duan, B. Stewart, M. Pu, J. Liu, T. Privalov, L. Sun, "Toward Controlling Water Oxidation Catalysis: Tunable Activity of Ruthenium Complexes With Axial Imidazole/DMSO Ligands" *J. Am. Chem. Soc.* **2012**, *134*, 18868-18880.

[31] L. Duan, Y. Xu, P. Zhang, M. Wang, L. Sun, "Visible Light-Driven Water Oxidation by a Molecular Ruthenium Catalyst in Homogeneous System" *Inorg. Chem.* **2010**, *49*, 209-215.

[32] L. Duan, F. Bozoglian, S. Mandal, B. Stewart, T. Privalov, A. Llobet, L. Sun, "A Molecular Ruthenium Catalyst with Water-Oxidation Activity Comparable to That of Photosystem II" *Nat. Chem.* **2012**, *4*, 418-423.

[33] L. Wang, D. W. Shaffer, G. F. Manbeck, D. E. Polyansky, J. J. Concepcion, "High-Redox-Potential Chromophores for Visible-Light-Driven Water Oxidation at Low pH" *ACS Catal.* **2019**, *10*, 580-585.

[34] N. Noll, A.-M. Krause, F. Beuerle, F. Würthner, "Enzyme-Like Water Preorganization in a Synthetic Molecular Cleft for Homogeneous Water Oxidation Catalysis" *Nat. Catal.* **2022**, *5*, 867-877.

[35] G. Das, D. A. P. Friedewald, D. Tang, K. Shoyama, F. Creazzo, S. Luber, F. Würthner, "Secondary Coordination Sphere Effects in Macrocycle-Embedded Mononuclear Ru(bda) Water Oxidation Catalysts" *Inorg. Chem.* **2026**, *65*, 4710–4720.

[36] M. Schilling, R. A. Cunha, S. Luber, "Zooming in on the O–O Bond Formation—An Ab Initio Molecular Dynamics Study Applying Enhanced Sampling Techniques" *J. Chem. Theory Comput.* **2020**, *16*, 2436-2449.

[37] M. Schilling, R. A. Cunha, S. Luber, "Enhanced Ab Initio Molecular Dynamics Exploration Unveils the Complex Role of Different Intramolecular Bases on the Water Nucleophilic Attack Mechanism" *ACS Catal.* **2020**, *10*, 7657-7667.

[38] R. Han, S. Luber, "Complete Active Space Analysis of a Reaction Pathway: Investigation of the Oxygen–Oxygen Bond Formation" *J. Comput. Chem.* **2020**, *41*, 1586-1597.

[39] F. H. Hodel, P. Deglmann, S. Luber, "Exploring Solvation Effects in Ligand-Exchange Reactions via Static and Dynamic Methods" *J. Chem. Theory Comput.* **2017**, *13*, 3348-3358.
